# Neuroimaging for prognosis of central nervous system infections: a systematic review and meta-analysis

**DOI:** 10.1186/s13613-025-01516-1

**Published:** 2025-07-16

**Authors:** Augustin Gaudemer, Netanel Covier, Marie-Cécile Henry-Feugeas, Jean-François Timsit, Philippa Catherine Lavallée, Etienne de Montmollin, Augustin Lecler, Antoine Khalil, Romain Sonneville, Camille Couffignal

**Affiliations:** 1 Department of Radiology, Hôpital Bichat Claude Bernard, AP-HP, Paris, France; 2 Université Paris Cité, INSERM, UMR 1137-IAME, Paris, France; 3 Department of Intensive Care, Hôpital Bichat Claude Bernard, AP-HP, 46 rue Henri Huchard, Paris Cedex, 75877 France; 4 Department of Neurology, Hôpital Bichat Claude Bernard, AP-HP, Paris, France; 5 Department of Neuroradiology, Fondation Adolphe de Rotschild, Paris, France; 6 URC, Hôpital Bichat Claude Bernard, AP-HP, Paris, France

**Keywords:** CNS infection, Neuroimaging, Meta-analysis, Functional outcome, Mortality, Encephalitis, Intensive care, Autoimmune, Outcome

## Abstract

**Background:**

Central nervous system (CNS) infections carry a severe prognosis and often require intensive care unit (ICU) admission. This study evaluated the prognostic value of neuroimaging in patients with all-type CNS infections.

**Methods:**

Using a predefined strategy, we first conducted a systematic search of PubMed/MEDLINE, PubMed Central, Embase, Cochrane and Google Scholar. Eligible studies published between January 1st, 2000, and June 1st, 2023, were included. We considered randomized controlled trials, non-randomized trials, cohort studies, excluding abstracts, cost-effectiveness analyses, letters, conference proceedings, systematic reviews, and meta-analyses. Two authors independently screened publications and extracted data. The meta-analysis was performed using a random-effects model. The main outcomes were (1) unfavorable outcome, defined as severe functional disability or death, and (2) mortality. Pooled odds ratios (OR) and 95% confidence intervals (95%CI) were calculated for each neuroimaging feature. We performed prespecified subgroup analyses depending on type of CNS infection (bacterial meningitis, CNS tuberculosis, CNS cryptococcosis, viral encephalitis, and brain abscess), country income, and ICU admission status.

**Results:**

Of 7,864 studies identified, 83 met the inclusion criteria, with 48 studies (6,434 patients) included in the meta-analysis. Abnormal MRI (OR: 3.55; 95%CI: 1.81–6.96; I²=0%), brain ischemia (OR: 4.65; 95%CI: 3.14–6.88; I²=28.5%), and hydrocephalus (OR: 4.56; 95%CI: 2.49–8.36; I²=61.5%) were significantly associated with unfavorable outcome. Hydrocephalus (OR, 3.99; 95%CI 1.83–8.70; I²=61%) and brain ischemia (OR, 3.51; 95%CI, 2.22–5.54; I²=16.4%) were associated with mortality. These associations remained consistent in patients with bacterial meningitis and in patients with CNS tuberculosis, but not in other CNS infections. Subgroup analyses depending on country income and ICU admission status revealed similar findings.

**Conclusion:**

Neuroimaging provides essential prognostic information in patients with CNS infections. Abnormal MRI findings, cerebral ischemia, and hydrocephalus are associated with unfavorable outcome, particularly in bacterial meningitis and CNS tuberculosis. These neuroimaging features should be considered when discussing prognosis in affected patients.

**Supplementary Information:**

The online version contains supplementary material available at 10.1186/s13613-025-01516-1.

## Background

Neuroimaging is widely employed throughout various central nervous system (CNS) processes, particularly in the context of CNS infections, serving as both a diagnostic tool and a means to identify cerebral complications. In patients with meningitis who present with focal neurological deficits, new-onset seizures, severely altered mental status, or clinical signs suggestive of brain herniation, neuroimaging is recommended to exclude a CNS process that may contraindicate lumbar puncture and/or to identify intracranial complications [1–3]. In patients with encephalitis, MRI is mandatory to rule out common confounders, and to identify patterns associated with specific infectious or autoimmune causes [4].

CNS infections are associated with a severe prognosis, especially among patients requiring care in an intensive care unit (ICU), with mortality rates up to 25% at three months, and significant disability in one third of survivors [5]. In this context, acquiring a more thorough comprehension of prognosis holds the potential to furnish precise information to both patients and their families, and customize medical interventions accordingly.

Neuroimaging has been suggested to assist with prognosis in specific situations, such as post-cardiac arrest patients [6–8], stroke [9–11], or traumatic brain injury [12–14]. In post-cardiac arrest patients, MRI has become an integral component of the recommended multimodal assessment, enabling physicians to estimate, as accurately as possible, the prognosis of each patient [15].

In severe meningitis and encephalitis patients, predictors of unfavorable outcomes include older age, immunodepression, neurologic presentation, and respiratory/cardiovascular complications [5]. While previous studies highlight brain imaging’s potential predictive value in *Herpes simplex* encephalitis [16, 17], its relevance in all-cause meningitis and encephalitis is unclear.

Literature about prognosis of CNS infections being heterogeneous and sparse, we chose to use meta-analysis as a tool to look for imaging prognosis factors. Thus, we aimed to synthesize available data and to pinpoint markers associated with functional outcome, mortality, and brain ischemia. We opted to assess CNS infections globally, despite the potential variation in neuroimaging patterns among different pathogens. Subsequently, we sought to identify distinct neuroimaging indicators for prognosis among common causes of CNS infections.

## Methods

### Search strategy and data sources

We conducted this systematic review according to the PRISMA reporting guidelines [18]. and we registered the protocol in PROSPERO (ID CRD42023385567). We searched eligible studies through PubMed/MEDLINE, Embase, Cochrane and Google Scholar. For the last grey database we chose to only look on the first ten pages to limit heterogeneity. The search key is available in supplementary (S1). We included studies published between January 1st, 2000 and June 1st, 2023.

### Eligibility criteria and outcomes

All studies focusing on patients with acute CNS infection and baseline brain imaging, were eligible for inclusion, between January 1st, 2000 and June 30th, 2023. High levels of heterogeneity due to multiple infections type, outcomes and study design were expected. We included randomized controlled trials, non-randomized trials, prospective and retrospective cohort studies, case-cohort studies, nested case control studies with more than 10 patients written in English. We excluded abstracts, cost effectiveness studies, letters, conference proceedings, systematic reviews, and meta-analyses. We also excluded pediatric studies (mainly patients with age < 15 years) because of the heterogeneity induced by the specific infections in children. Finally, we excluded studies conducted among patients with COVID-19 due to lack of hindsight (< 5 years) and different mechanisms of CNS injury (mostly vascular and microvascular). Research key was elaborated in this purpose (table S1).

Exposure and control were defined as the abnormality of neuroimaging or not respectively (table S2). Functional outcomes mostly included modified Rankin Scale (mRS), Glasgow Outcome Scale (GOS), Glasgow Outcome Scale Extended (GOSE), Modified Barthel Index (MBI). Each patient was classified as favorable or unfavorable outcome as they were in original publications (i.e. mRS ≥ 3, GOS ≤ 3–4, MBI ≤ 12) [19–23]. We also looked for prognosis neuroimaging marker of mortality and brain ischemia if available.

### Data extraction

Two authors (AG and MCHF) screened eligible studies independently, using Rayyan [24]. Discrepancies were resolved by an independent decision from a third author (RS). A Microsoft excel sheet was used independently by two authors (AG and NC) to extract data from reports, such as study design, patient’s characteristics, and neuroimaging features, e.g. ischemia, hydrocephalus or other ventricular abnormalities, leptomeningeal enhancement, vascular enhancement, diffusion abnormalities. “Abnormal MRI” was noted when no further details were available or when it was independently studied in publications.

### Evidence of study quality

We adapted a scale for our study from Risk of Bias Assessment Tool for Nonrandomized Studies [25] Newcastle-Ottawa Scale [26] with imaging criteria (Table S3). Imaging quality criteria included a reproductible imaging protocol and a dedicated and specialized neuroimaging analysis blinded from clinical data. Each study had a score between 0 and 11, with higher scores indicating better quality for imaging analysis.

### Statistical analysis

Potential prognosis markers were studied using the package metafor version 4.2-0 on R software version 2023.03.0. Pooled odds ratios (OR) and their 95% confidence intervals (CI) were estimated using a random-effect model. Each study was weighted on the quality score and number of patients included. Between-study heterogeneity was assessed for each analysis, with *p* < 0.1 or I²>50% indicating significant heterogeneity. Publication bias was assessed with a funnel plot.

## Results

### Literature search

**Fig. 1:**
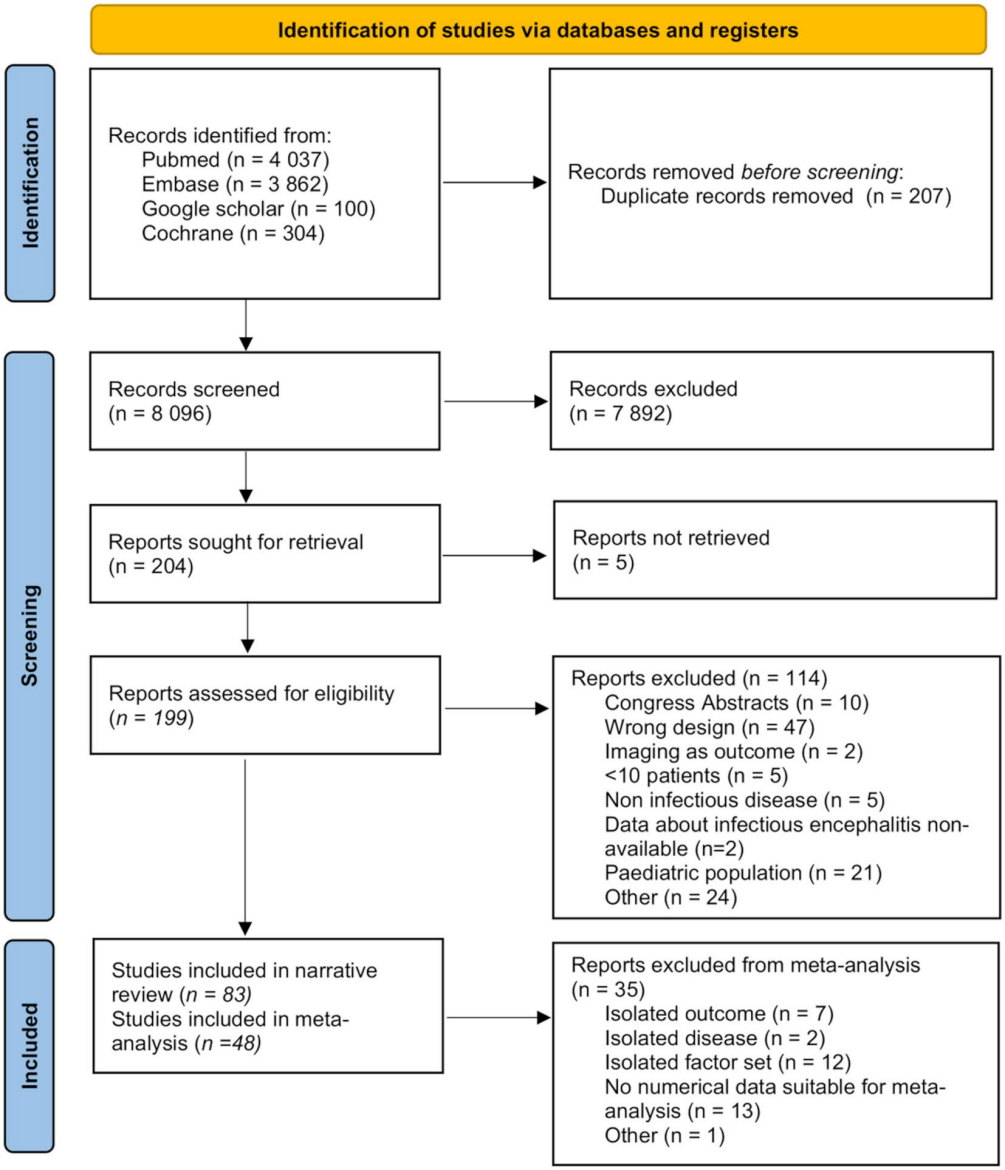
PRISMA diagram of eligible studies

A total of 7864 records were screened. Eighty-three studies were included in the systematic review and 48 studies in the meta-analysis with a combined total of 6434 patients. This corresponded to a median of 90 (interquartile range, 45–116) patients per study. Details of exclusion at each step is available on flow-chart (Fig. 1).

### Study characteristics

Among 83 studies included in the systematic review, 7 were about bacterial meningitis, 12 about fungal meningitis (i.e. cryptococcal meningitis, *n* = 9; aspergillosis, *n* = 2; coccidioidal meningitis, *n* = 1), 35 about CNS tuberculosis, 10 about brain abscesses, 14 about viral meningitis or encephalitis (including *n* = 8 about *Herpes simplex* virus encephalitis), and 5 miscellaneous or including multiple infectious agents (Table 1). The median time between admission or diagnosis and baseline imaging, which was available for 7 of 83 studies, was 1.5 days (interquartile range, 1–2 days). The median of quality assessment score was 7 (interquartile range 5.75-8). A detailed assessment of each study is available on supplementary material (Table S4).

**Table 1:** Characteristics of the 86 included studies in the systematic review. *quality index as a score between 0 and 11, with higher scores indicating better quality for imaging analysis based on risk of NOS (Stang, Eur J epidemiol. 2013) and RoBANS (Kim, J Clin Epidemiol. 2013). Typologies included radiological quality criteria (double reading, blinding from clinical data, dedicated and systematic acquisition protocol). Distribution of quality index was normal

Variable		*N* = 86
**Year of first inclusion**		2004 [1983–2019]
**Year of last inclusion**		2013 [1996–2022]
**Country**		
	India	21 (25%)
	China	14 (17%)
	Taiwan	10 (12%)
	France	7 (8%)
	Korea	4 (5%)
	USA	4 (5%)
	Pakistan	4 (5%)
	Turkey	4 (5%)
**Study design**		
	Prospective cohort study	59 (71%)
	Retrospective cohort study	23 (28%)
	Randomized controlled trial	1 (1%)
**CNS infection type**		
	CNS tuberculosis	35 (42%)
	Viral encephalitis	14 (17%)
	Acute fungal meningitis	12 (15%)
	Brain abscess	10 (12%)
	Acute bacterial meningitis	7 (8%)
	Acute infectious meningitis (multiple agents)	3 (4%)
	Fungal encephalitis	1 (1%)
	Infectious encephalitis (multiple agents)	1 (1%)
**Functional outcome**		
	modified Rankin Scale ≥ 3	15 (18%)
	Modified Barthel Index ≤ 12	12 (14%)
	Glasgow Outcome Scale ≤ 4	6 (7%)
	Glasgow Outcome Scale ≤ 3	6 (7%)
	Glasgow Outcome Scale Extended ≤ 4	1 (1%)
**Patients per study**		81 [45–114]
**MRI per study**		71 [41–108]
**CT per study**		80 [36–123]
**MRI per patient per study**		1 [0.85 − 1]
**Most evaluated neuroimaging factor sets among studies**		
	Hydrocephalus	33 (40%)
	Ischemia	19 (23%)
	Abnormal MRI	9 (11%)
Median quality index (/11)*		7 [5.75–8]

*Data are median* [Q1 – Q3] or number (percentage)

*Quality index ranges as a score between 0 and 11,* with higher scores indicating better quality for imaging analysis (based on references* [25, 26])

Data on the immunocompromised status of patients were available in 21 studies. Five hundred and six patients were reported to have altered immune status, including 184 HIV-positive patients. No specific imaging data were available for this population, preventing further analysis.

### Outcome analyses

#### Functional outcome

**Fig. 2:**
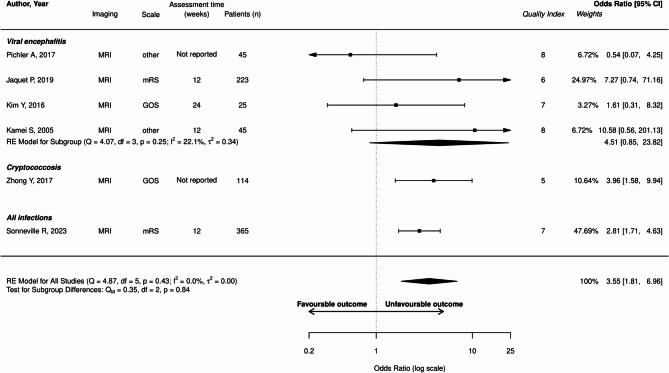
Forest plot illustrating the relationship between abnormal MRI findings and unfavorable outcomes across included studies. “Quality index” refers to the quality scale adapted from NOS and RoBANS. Detailed description for each study is available in supplemental material. mRS, modified Rankin Score; GOS, Glasgow Outcome Scale; MBI, modified Barthel Index; CI, confidence interval; df, degrees of freedom; IV, inverse variance; Q, Q-statistic; Qm, Qm-statistic. Sonneville et al. 2023 [5], Sarton et al. 2021 [17], Jaquet et al. 2019 [16], Pichler et al. 2017 [27], Kim et al. 2016 [28], Kamei S. et al. 2005 [29], Zhong Y et al. 2017 [30]

**Fig. 3:**
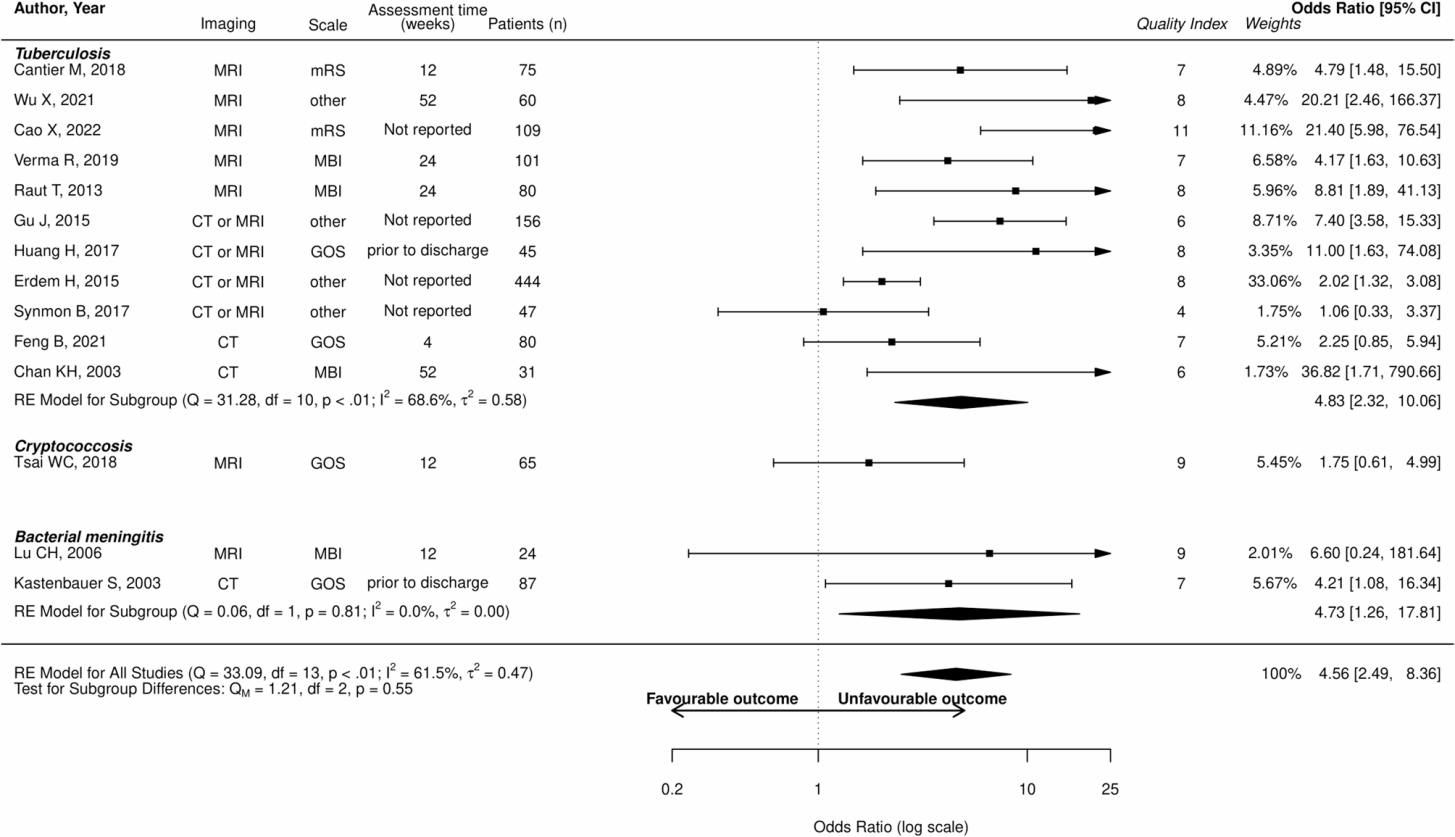
Forest plot illustrating the relationship between hydrocephalus and unfavorable outcomes across included studies. “Quality index” refers to the quality scale adapted from NOS and RoBANS. Detailed description for each study is available in supplemental material. mRS, modified Rankin Score; GOS, Glasgow Outcome Scale; MBI, modified Barthel Index; CI, confidence interval; df, degrees of freedom; IV, inverse variance; Q, Q-statistic; Qm, Qm-statistic. Wu X et al. 2021 [31], Gu et al. 2015 [32], Cao et al., 2022 [33], Verma et al., 2019 [34], Raut et al., 2013 [35], Chan et al. 2003 [36], Feng et al. 2021 [37], Huang et al. 2017 [38], Erdem et al. 2015 [39], Synmon et al. 2017 [40], Tsai et al. 2018 [41], Lu et al. 2006 [42], Kastenbauer et al. 2003 [43]

**Fig. 4:**
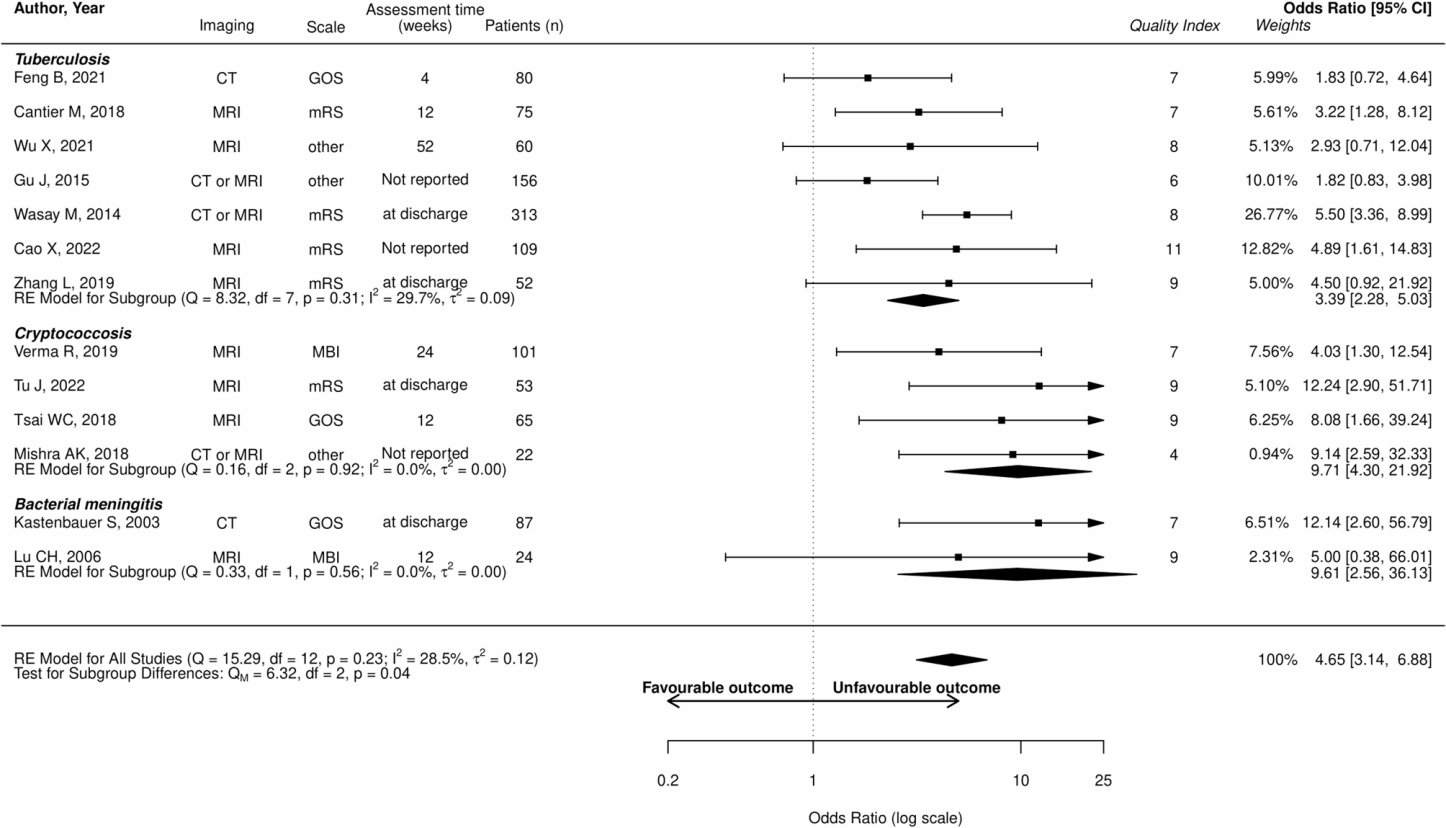
Forest plot illustrating the relationship between brain ischemia and unfavorable outcomes across included studies. “Quality index” refers to the quality scale adapted from NOS and RoBANS. Detailed description for each study is available in supplemental materia. mRS, modified Rankin Score; GOS, Glasgow Outcome Scale; MBI, modified Barthel Index; CI, confidence interval; df, degrees of freedom; IV, inverse variance; Q, Q-statistic; Qm, Qm-statistic. Cao et al. 2022 [33], Wu et al. 2021 [31], Feng et al. 2021 [37], Zhang et al. 2019 [44], Verma et al. 2019 [34], Cantier et al. 2018 [45], Gu et al. 2015 [32], Wasay et al. 2014 [46], Tu et al. 2022 [47], Mishra et al. 2018 [48], Tsai et al. 2018 [41], Lu et al. 2006 [42], Kastenbauer et al. 2003 [43]

The proportion of unfavorable outcome in the whole population was 36.8% (2901/7873 patients). Abnormal MRI was associated with unfavorable outcome (OR: 3.55; 95%CI: 1.81–6.96; I²=0%, Fig. 2). Hydrocephalus (OR: 4.56; 95%CI: 2.49–8.36; I²=61.5%) and brain ischemia (OR: 4.65; 95%CI, 3.14–6.88; I² 28.5%) were also associated with unfavorable outcome (Figs. 3 and 4).

#### Mortality

Mortality in the whole population was 18.7% (644/3447 patients). Hydrocephalus (OR, 3.99; 95%CI 1.83–8.70; I²=61%; figure S1) and brain ischemia (OR, 3.51; 95%CI, 2.22–5.54; I²=16.4%) were associated with mortality (Figure S2).

#### All infections excluding tuberculosis

Tuberculosis was the most common infection studied in included publications (42,17% of patients, see Table 1). When excluding tuberculosis, hydrocephalus (OR: 3.13; 95%CI: 1.29–7.62; I² = 0%, figure S6) and brain ischemia (OR: 9.66; 95%CI: 4.32–21.63; I² = 0%, figure S7) were associated with unfavorable outcome. Hydrocephalus (OR: 4.07; 95%CI: 1.12–14.79; I² = 73.5%, figure S8) and brain ischemia (OR: 3.86; 95%CI: 1.24–12.01; I² = 51.4%, figure S9) were also associated with mortality.

### Specific CNS infections

#### Bacterial meningitis

Hydrocephalus (OR: 4.75; 95%CI: 1.26–17.81; I² = 0%) and brain ischemia (OR: 9.61; 95% CI: 2.56–36.13; I² = 0%) were associated with unfavorable outcome. Hydrocephalus was not significantly associated with mortality (OR: 1.88; 95%CI: 0.52 − 6.8; I² =20.3%). One study showed an association between brain ischemia and mortality (OR: 6.37; 95%CI: 1.68–24.86) [49].

#### Viral encephalitis

Bilateral involvement (OR: 4.33; 95%CI: 2.11–8.90; I²=11.4%) and restricted diffusion (OR: 3.95; 95%CI: 1.57–9.90; I²=0%) were associated with poor functional outcomes (Figures S10-S11). Extensive involvement was not associated with functional outcome (OR: 5.71; 95%CI: 0.56-58.52; I² =86.2%, Figure S12). Two studies showed an association between hemorrhage and unfavorable outcome, without poolable data [17, 50].

#### CNS tuberculosis

Hydrocephalus (OR: 4.56; 95%CI: 2.15–9.65; I²=29.7%) and brain ischemia (OR: 3.39; 95%CI: 2.28–5.03; I²=68.6%) were associated with unfavorable outcome. Basal exudates were associated with unfavorable outcome (OR: 2.2; 95%CI: 1.07–4.68; I²=60.3%, Figure S13). Tuberculomas (OR: 1.50; 95%CI: 0.70 − 3.20; I² = 80.5%), abscesses (OR: 1.96; 95%CI: 0.75-5.12; I²=27.4%) and vasculitis (OR: 5.59; 95%CI: 0.24-128.15; I²=92.8%) were not associated with unfavorable outcome (data not shown). Hydrocephalus (OR: 3.79; 95%CI, 2-7.16; I²=0%) and brain ischemia (OR: 3.32; 95%CI, 2.27–6.4; I²=0%) were associated with mortality.

#### Cryptococcosis

Abnormal MRI was associated with unfavorable outcome in one study (OR: 3.96; 95%CI, 1.58–9.94) [30]. Hydrocephalus was not associated with unfavorable outcome in another study (OR: 1.75; 95%CI: 0.61-4.99) [41]. Brain ischemia was associated with unfavorable outcome (OR: 9.71; 95%CI: 4.30-21.92; I²=0%). Neither hydrocephalus (OR: 3.26; 95%CI: 0.35-3.04; I²=70.5%), nor brain ischemia (OR: 1.17; 95% CI: 0.43-3.24; I²=0%) were not associated with mortality.

#### Brain abscess

Multiple abscesses (OR: 1.47; 95%CI: 0.72 − 3.00; I²=0%, Figure S14) and intraventricular rupture (OR: 1.5; 95%CI: 0.31-7.21; I²=86.9%, Figure S15) were not associated with unfavorable outcome.

Data about other neuroimaging features and other CNS infection is available on supplementary materials (table S3).

### Low-income vs. high-income countries

There were no significant differences reported between low-income and high-income countries regarding hydrocephalus, brain ischemia, and abnormal MRIs in terms of functional outcomes or mortality. However, a lower odds ratio (OR) for mortality in brain ischemia was observed in high-income countries (p-value, 0.08). Forest plots with subgroup analysis based on national income are available in supplementary materials (Figures S16 to S20).

### ICU vs. non-ICU patients

No significant differences were found between ICU patients, non-ICU patients and unknown or mixed origin patient (Figures S21 - S23) for abnormal MRI (p-value, 0.25), hydrocephalus (p-value, 0.36) or brain ischemia (p-value, 0.68).

### Publication bias

For associations between outcome and main neuroimaging features, diagnostic plots evaluating distribution were performed and available in supplemental material (figures S3-S5).

## Discussion

In this large systematic review and analysis of 83 studies encompassing 6434 adult patients with CNS infections, we identified several neuroimaging variables associated with unfavorable outcome, in the form of severe disability or death. Specifically, abnormal MRI, hydrocephalus, and brain ischemia, were found to be associated with unfavorable outcome. Hydrocephalus and brain ischemia were associated with mortality. All these neuroimaging variables were identified from widely available sequences performed in routine practice.

To our knowledge, our meta-analysis stands as the most extensive examination published on the prognostic significance of neuroimaging in acute CNS infections and fills an important knowledge gap. It is important to note that CNS infections comprise a wide range of illnesses, involving diverse viruses, bacteria, and other microorganisms. Existing literature, particularly meta-analyses, has predominantly focused on the diagnostic value of neuroimaging features [51, 52] of specific types of CNS infection, such as viral encephalitis [53]. When assessed, the prognostic significance of neuroimaging features observed in these studies was limited to a particular subtype of CNS infection. A major strength of our study was to consider all-cause CNS infections in the main analysis, irrespective of the specific microorganisms involved. This approach allowed us to aggregate several diseases based on clinical presentation, severity, and neuroimaging features rather than hospital discharge diagnosis. This approach parallels with clinical practice, where neuroimaging is often conducted early in the course of illness, often before any microbiological diagnosis has been made.

We observed substantial heterogeneity in our results. The prognostic value of neuroimaging variable varied across different types of CNS infections, and there were instances where specific data were unavailable. For instance, among patients with bacterial meningitis, hydrocephalus was associated with an unfavorable outcome, but not with mortality. Because neuroimaging is not routinely performed in non-severe cases of bacterial meningitis, there is a scarcity of data regarding neuroimaging, which might account for the disparity between unfavorable outcome and mortality rates. Diffuse abnormalities such as subarachnoid hemorrhage, diffuse brain swelling, or extensive leptomeningeal enhancement were previously described as markers of unfavorable outcome [43, 54–57], but we were unable to find sufficient data for a numerical analysis. It is also worth noting that very uncommon complications, such as hydrocephalus and vasculitis in *Herpes simplex* encephalitis have only been documented in case reports, which were excluded from our analysis [58–60]. Despite their rarity, these complications might also serve as potential indicators of adverse outcome.

This strong heterogeneity prompted us to conduct sensitivity analyses to explore each potential confounding factor. Interestingly, no significant differences were observed between patients from ICU departments and those from other settings. It is possible that more severely ill ICU patients were too unstable to undergo brain imaging, which may have led to an underestimation of the prognostic value of this imaging.

We did not observe any significant differences between low- and high-income countries. We only showed a tendency to a lower OR in high income countries for mortality when patients presented a brain ischemia. It could be partly explained by two factors. First, CNS infections were different between low and high-income countries. Especially, no study about acute bacterial meningitis was included in the high-income countries subgroup. Second, the standard of care may be different for brain ischemia with more neurovascular care in high-income countries.

We also performed subgroup analyses for main CNS infections. In CNS tuberculosis patients, hydrocephalus was associated with both unfavorable outcomes and mortality. This complication is a hallmark of CNS tuberculosis with a pathogenesis encompassing both obstructive (such as ventricular obstruction by a granuloma) and non-obstructive (involvement of Pacchioni granulations) mechanisms [61]. While hydrocephalus may manifest overtly, it sometimes coexists with brain atrophy, potentially leading to misdiagnosis.

Brain ischemia showed a strong association with unfavorable outcomes and mortality, as well as a correlation with basal exudates in CNS tuberculosis patients, suggesting a link with vasculitis pathogenesis [62]. Gelatinous exudates may contribute to intimal thickening and obliterative vasculitis, particularly at the base of the brain. Despite this, no significant association was found between vasculitis and unfavorable outcomes, possibly due to the lack of a standardized neuroimaging definition.

Intraventricular rupture of brain abscesses or multiple abscess locations did not significantly associate with unfavorable outcome, likely influenced by limited data availability. Although a previous study indicated a significant association between ventriculitis and multiple abscesses [63], data suitable for pooling were lacking. Finally, similarities in findings between CNS cryptococcosis and tuberculosis, particularly regarding hydrocephalus, brain ischemia, and basal exudates, suggest that specific leptomeningeal and anatomical involvement may lead to similar complications, such as obstructive and non-obstructive hydrocephalus.

Surprisingly, an impairment over 3 lobes in viral in encephalitis was not associated with a unfavorable outcome [16, 17, 64], but bilateral involvement was. This might be inherent to small sample and methodological heterogeneity. A volumetric approach using supervised segmentation on FLAIR T2 or diffusion-weighted imaging sequences may offer greater accuracy in this type of evaluation. To date, no study has assessed this approach in CNS infections.

Only a limited number of studies have provided data regarding the interval between admission or diagnosis and neuroimaging. It is widely recognized that delayed diagnosis and treatment are predictive of unfavorable outcomes [5]. These results once again underscore the necessity to standardize clinical practice concerning the timing of neuroimaging in these infections.

Data on mortality were limited and heterogeneous. Most of the included studies were not primarily designed to assess prognosis or identify potential prognostic markers, and key outcome data were often missing. This limitation is partly inherent to the methodological choices of our review. Nonetheless, synthesizing the available data may still offer preliminary insights into possible associations between imaging findings and mortality. However, well-designed prospective studies are needed to evaluate the prognostic value of these imaging features more accurately.

No data on potential adverse events during MRI examinations was available in the included studies. However, a recent study involving 4,434 ICU patients who underwent MRI found only one severe adverse event (0.3%) [65]. Multiple potential risk factors for adverse events were described previously, including patient-related factors such as comorbidities or intubation, and operational factors such as the distance to the imaging department or a lack of communication [66]. These findings highlight the need for a thorough evaluation of the benefits and risks before undergoing such examinations.

Diffusion-weighted imaging was identified as a prognostic factor in Herpes simplex encephalitis [17]. However, it is primarily used as a diagnostic tool, aiming to differentiate infectious from auto-immune encephalitis [67]. Diffusion weighted imaging has not been further studied in CNS infections, and suffers from a lack of standardization. Indeed, diffusion alteration such as restriction reflects cytotoxic edema, but no data processing has been standardized, such as apparent diffusion coefficient cut-off or degrees of extension. A clearer and well-defined volumetric or regional data approach to diffusion MRI data, similar to that used in post-cardiac arrest syndrome neuro-prognostication [8, 68, 69] could be valuable for a more comprehensive understanding of the significance of this factor. This lack of standardization may explain why it could not be included in our meta-analysis.

Altogether, our study underscores the need for the standardization of clinical practices concerning neuroimaging in CNS infections and, perhaps, a more systematic approach to its interpretation with clearer definitions of neuroimaging features. Furthermore, this encompasses not only the need for addressing technical issues but also determining the ideal timing of neuroimaging within standard care.

Our study has several strengths. Our large meta-analysis seems to fill a knowledge gap-to date, no meta-analysis was conducted about prognostic neuroimaging features of CNS infection. We chose to apprehend CNS infection as a clinical syndromic entity instead of etiological entity. It allowed us to apprehend neuroimaging features not as a neuroradiological syndrome but as isolated elementary lesions. These lesions were groupable even if germs and mechanisms were different.

Our study has also limitations. We expected a high level of heterogeneity in this analysis. Firstly, CNS infections have quite different pathophysiology, and are associated with different imaging patterns, particularly in terms of extension. However, despite different pathophysiological pathways, hydrocephalus and ischemia appear to be common complications of different infections, possibly justifying their use as prognostic markers.

Most studies also did not have imaging evaluation as their primary objective. Their design generally did not include standardization of imaging, either in terms of practical execution (no harmonization of protocols) or interpretation (no specialized review). This is obviously linked to the absence of global recommendations on imaging CNS infections. However, this approach, which considers the heterogeneity of the literature, reflects the reality of daily practice, and probably brings our results closer to that reality.

A second area of considerable heterogeneity in our study was the small number of studies and patients. From a statistical point of view, this reduced the possibility of studying classic markers such as I². Indeed, some I²s were at 0%, with very wide related confidence intervals linked to these small sample sizes. This has already been reported as a major limitation to the interpretation of these tools [70, 71].

## Conclusion

Our study shows that neuroimaging provides essential prognostic information in patients with CNS infections. Abnormal MRI findings, cerebral ischemia, and hydrocephalus are associated with unfavorable outcomes, particularly in bacterial meningitis and CNS tuberculosis. These neuroimaging features should be considered when discussing prognosis in affected patients.

## Electronic supplementary material

Below is the link to the electronic supplementary material.

Supplementary Material 1

Supplementary Material 2

## Data Availability

Data and material will be made available on reasonable request.

## Abbreviations

CNS: central nervous system
MRI: magnetic resonance imaging
CT: computed tomography
ICU: Intensive Care Unit
mRS: modified Rankin Score
GOS: Glasgow Outcome Scale
MBI: modified Barthel Index
CSF: Cerebrospinal Fluid
ICU: Intensive Care Unit
HIV: Human Immunodeficiency Virus
GCS: Glasgow Coma Scale
AIDS: Acquired Immunodeficiency Syndrome
MERS: Mild Encephalopathy with Reversible Splenial Lesion
DWI: Diffusion-Weighted Imaging
ADC: Apparent Diffusion Coefficient
T2WI: T2-Weighted Imaging
T1WI: T1-Weighted Imaging
FLAIR: Fluid-Attenuated Inversion Recovery
ICD: International Classification of Diseases
NOS: Newcastle Ottawa Scale
