# Supplementary material for: Neuroimaging for prognosis of central nervous system infections: a systematic review and meta-analysis
Source: Ann Intensive Care. 2025 Jul 16;15:101. doi: 10.1186/s13613-025-01516-1 (PMC12267735; doi:10.1186/s13613-025-01516-1)
Supplement: Supplementary file 2 — Supplementary Material 2 [file 13613_2025_1516_MOESM2_ESM.docx]

**Neuroimaging for prognosis of central nervous system infections: a systematic review and meta-analysis**

Augustin Gaudemer (M.D.)^1,2^, Netanel Covier(M.D.)^1^, Marie-Cécile Henry-Feugeas (M.D., Ph.D.)^1^, Jean-François Timsit (M.D., Ph.D.)^2,3^, Philippa Lavallée (M.D, Ph.D.)^4^, Augustin Lecler (M.D, Ph.D)^5^, Etienne de Montmollin (M.D., Ph.D.) ^2,3^, Antoine Khalil (M.D., Ph.D.)^1^, Romain Sonneville (M.D., Ph.D.)^2,3,#^, Camille Couffignal (PharmD., Ph.D.)^2,6,#^

1. Department of Radiology, Hôpital Bichat Claude Bernard, AP-HP, Paris, France

2. Université Paris Cité, INSERM, UMR 1137-IAME, Paris, France

3. Department of Intensive Care, Hôpital Bichat Claude Bernard, AP-HP, Paris, France

4. Department of Neurology, , Hôpital Bichat Claude Bernard, AP-HP, Paris, France

5. Department of Neuroradiology, Fondation Adolphe de Rotschild, Paris, France

6. URC, Hôpital Bichat Claude Bernard, AP-HP, Paris, France

^#^authors equally contributed to this work

**Corresponding author:** Prof. Romain Sonneville, M.D., Ph.D.

Service de médecine intensive - réanimation

Hôpital Bichat – Claude Bernard, 46 rue Henri Huchard, 75877 Paris Cedex, France.

E-mail: romain.sonneville@aphp.fr; Tel.: +33-1-40257702; Fax: +33-1-40258782.

Table S1. Pubmed Key.

(brain[TW] OR brains[TW] OR brainstem*[TW] OR head[TW] OR heads[TW] OR craniocerebral*[TW] OR intracrani*[TW] OR intra-crani*[TW] OR cerebr*[TW] OR cerebel*[TW] OR forebrain*[TW] OR brain[MH])

AND (abscess[TW] OR encephalitis[TW] OR meningitis[TW] OR meningiditis[TW] OR infection[TW] OR "Infectious Encephalitis"[MH] OR "Meningitis"[MH] OR "brain inflammation*"[TW] OR "choriomeningiti*"[TW] OR "Arachnoiditi*"[TW])

AND (magnetic*[TW] OR “Magnetic Resonance Imaging”[TW] OR "diffusion weighted"[TW] OR "diffusion tensor"[TW] OR MRI[TW] OR MR[TW] OR fMRI [TW] OR dMRI[TW] OR MRS[TW] OR MRA[TW] OR DTI[TW] OR DWI[TW] OR "T1-weighted"[TW] OR "T1 weighted"[TW] OR T1WI[TW] OR T1[TW] OR T1rho[TW] OR "T2-weighted"[TW] OR "T2 weighted"[TW] OR T2WI[TW] OR T2[TW] OR "T2-weighted"[TW] OR "T2WI"[TW] OR "T2"[TW] OR "T2-Gradient Echo"[TW] OR "T2-GRE"[TW] OR "Fluid attenuated inversion recovery"[TW] OR FLAIR[TW] OR "Susceptibility weighted"[TW] OR SWI[TW] OR SWAN[TW] OR "Magnetic Resonance Imaging"[MH] OR "Diffusion Magnetic Resonance Imaging"[MH:Exp] OR spectroscopy[TW] OR SRM[TW] OR "Proton Magnetic Resonance Spectroscopy"[MH] OR "arterial spin labelling"[TW] OR "ASL"[TW] OR "pcasl"[TW] OR “DSC”[TW] OR “dynamic susceptibility”[TW] OR “perfusion” OR computed tomog*[TW] OR ct[TW] OR cta[TW] OR "Tomography, X-Ray Computed"[MH])

AND (Incidence[MH:NoExp] OR Mortality[MH] OR Follow Up Studies[MH:NoExp] OR prognos*[TW] OR predict*[TW] OR course*[TW] OR prognosis[MH] OR multicent*[TW] OR "pronostic*"[TW] OR “outcome*”[TW] OR *functional*[TW])

NOT (animals [MH] NOT humans [MH])

NOT ("neoplasms"[Majr])

NOT ("case report"[TI] OR "case reports"[Publication Type] NOT ("case series*"[TI] OR "case series"[OT]))

AND (English[LA] OR French[LA])

AND ("2000/01/01"[Date - Publication] : "2023/03/15"[Date - Publication])

Table S2. PECO for main analysis.

| Population | All patients hospitalized with infectious meningitis or meningo-encephalitis who underwent brain imaging (CT and/or MRI) |
| --- | --- |
| Exposure | Abnormal imaging (CT or MRI, as defined as authors), hydrocephalus on imaging, brain ischemia on imaging |
| Control | Normal imaging, no hydrocephalus on imaging, no brain ischemia on imaging |
| Outcome | Unfavorable outcome (as defined by authors) |

Table S3. Quality scale, adapted from NOS and RoBANS.

| **Bias type** | **Low-risk bias** | **Unclear** | **High-risk bias** |
| --- | --- | --- | --- |
| **Selection of participant** | | | |
| **Selection of cases or experimental/interventional group or for cohort unique group or exposed group*** | Method of recruitment and participant selection is clearly described.   Study participants were consecutively recruited. | It is uncertain whether the selection of participants results in a ‘high risk’ or a ‘low risk’ bias. | It is unclear or not documented how participants were selected. Study participants were recruited using convenience sampling. |
| **Selection of controls or of RCT control/SOC group or for cohort non-exposed group*** | Method of control selection is clearly described.   Case-control, cohort : Control participant are recruited from the same population.  Study control were consecutively recruited, with same definition of infection and outcome. | It is uncertain whether the selection of controls results in a ‘high risk’ or a ‘low risk’ bias. | Case–control studies: favorable and unfavorable outcome patients are selected from different population groups (differing study center or historical control groups)  It is unclear or not documented how controls were selected. Study controls were recruited using convenience sampling. |
| **Definition of Controls*** | Clearly defined as good clinical outcome (based on mRANKIN, GOS) | It is uncertain whether the definition of controls results in a ‘high risk’ or a ‘low risk’ bias. | Other or no description |
| **Assessment of CNS infection** | CNS infection diagnosis relies on microbiological test.  CNS infection diagnosis methods is explicit. | It is uncertain whether the assessment of CNS infection results in a ‘high risk’ or a ‘low risk’ bias. | CNS infection diagnostics relies on imaging. |
| **Representativeness of the cases*** | Consecutive series of cases / a priori defined and justified period of collection data | it is unclear if cases were recruited consecutively or in an a priori and justified period. | potential for selection biases, or not stated |

| **Comparatibility** | | | |
| --- | --- | --- | --- |
| **Confounding variables: comparability of cases and controls on the basis of the design or analysis*** | No significant differences between both groups   Confounding variables are considered during analysis of results  Cohort studies: Study report appropriate information on participant background characteristics, in particular immunocompromised status | It is unclear if confounding variables were adequately considered. | Major confouding variables (i.e. significant differences on demographic or clinical presentation) are reported but not adequately considered during design and analysis phases  No consideration of immunocompromised status |
| **Exposure** | | | |
| **Method of Imaging (ROBANS) Same imaging method (NOS)*** | Imaging was dedicated to CNS infection outcome assessment  Imaging protocol was defined prior to study and was similar among patients | It is uncertain whether the method of imaging results in a ‘high risk’ or a ‘low risk’ bias. | Imaging conducted for other purpose and retrospectively evaluated |
| **Ascertainment of imaging data*** | Independent review of imaging data  Use of validated methods of measurement (e.g. volumetric analysis)  Adequate quality control of scan results (e.g. use of two independent radiology reports, report of interreporter reliability or quantitative analysis) | It is uncertain whether the ascertainment of imaging results in a ‘high risk’ or a ‘low risk’ bias. | Evaluation on imaging report, no dedicated analysis for study.  No documentation of who interpreted exams. |
| **Blinding of imaging assessments** | Scan reporters were blinded of outcome (or absence of blinding was judged to have no effect on the outcome measurements). | It is uncertain whether the blinding of imaging reporters results in a ‘high risk’ or a ‘low risk’ bias. | No documentation of blinding.  No blinding when it appears to affect the outcome measures. |

| **Incomplete or missing data management** | | | |
| --- | --- | --- | --- |
| **Incomplete data and selective outcome reporting** | No missing data.  All participants accounted for at conclusion of study.  The experimental protocol is available and the pre-defind primary and secondary outcomes were described as planned.  All of the expected outcomes were included in the study descriptions. | It is uncertain whether the selection of participants results in a ‘high risk’ or a ‘low risk’ bias. | Missing data could affect outcomes.  Participants lost to follow up were not accounted for.  The pre-defined outcomes were no fully reported.  Primary outcomes that were not pre-specified in the study existed.  Outcomes differed from previously defined standards.  The absence of reports on important outcomes that would be expected to be reported for studies in related fields. |
| ***items adapted from NOS** |  |  |  |

Table S4. Detailed quality assesment for included studies, adapted from NOS and RoBANS. Detailed criteria are available on table S2.

| **Author (1st)** | **Year** | Selection of cases or experimental / interventional group or for cohort unique group or exposed group* | Selection of controls or of RCT control/SOC group or for cohort non-exposed group* | Definition of Controls* | Assessment of CNS infection | Representativeness of the cases* | Confounding variables : comparability of cases and controls on the basis of the design or analysis* | Assesment of outcome | Method of Imaging (ROBANS) Same imaging method (NOS)* | Ascertainment of imaging data* | Blinding of imaging assessments | Incomplete data and selective outcome reporting | Quality index |
| --- | --- | --- | --- | --- | --- | --- | --- | --- | --- | --- | --- | --- | --- |
| Zheng H | 2015 | Low-risk | Low-risk | Low-risk | Low-risk | Low-risk | Low-risk | Low-risk | Unclear | Unclear | Unclear | Low-risk | 8 |
| Zhang L | 2019 | Low-risk | Low-risk | Low-risk | Low-risk | Unclear | Low-risk | Low-risk | Low-risk | Low-risk | Low-risk | Unclear | 9 |
| Sarton B | 2021 | Low-risk | Low-risk | Low-risk | Low-risk | Low-risk | Low-risk | Low-risk | Low-risk | Low-risk | Low-risk | Low-risk | 11 |
| Tan IL | 2012 | High-risk | Low-risk | Unclear | High-risk | Unclear | Unclear | Low-risk | Unclear | Unclear | Unclear | Unclear | 2 |
| Demir MK | 2007 | Unclear | Low-risk | Low-risk | Unclear | Unclear | Unclear | Low-risk | Low-risk | Low-risk | Unclear | Unclear | 5 |
| Tseng JH | 2006 | Low-risk | Low-risk | Low-risk | Low-risk | Low-risk | Low-risk | Low-risk | Unclear | Unclear | Unclear | Low-risk | 8 |
| Xiao F | 2005 | Low-risk | Low-risk | Low-risk | Low-risk | Low-risk | Low-risk | Low-risk | Unclear | Unclear | Unclear | Low-risk | 8 |
| Nathoo N | 2011 | Low-risk | Low-risk | Low-risk | Low-risk | Low-risk | Low-risk | Unclear | High-risk | Unclear | Unclear | Unclear | 6 |
| Sporrborn JL | 2015 | Low-risk | Low-risk | Low-risk | Low-risk | Low-risk | Low-risk | Low-risk | Low-risk | Unclear | Unclear | Unclear | 8 |
| Lu CH | 2006 | Low-risk | Low-risk | Low-risk | Low-risk | Low-risk | Low-risk | Low-risk | Low-risk | Unclear | Unclear | Low-risk | 9 |
| Cao X | 2022 | Low-risk | Low-risk | Low-risk | Low-risk | Low-risk | Low-risk | Low-risk | Low-risk | Low-risk | Low-risk | Low-risk | 11 |
| Wasay M | 2014 | Low-risk | Low-risk | Low-risk | Low-risk | Low-risk | Unclear | Low-risk | Unclear | Low-risk | Low-risk | Unclear | 8 |
| Tu J | 2022 | Low-risk | Low-risk | Low-risk | Low-risk | Low-risk | Low-risk | Low-risk | Low-risk | Low-risk | Unclear | Unclear | 9 |
| Hong D | 2013 | Low-risk | Low-risk | Low-risk | Unclear | Low-risk | Low-risk | Low-risk | Unclear | Unclear | Unclear | Low-risk | 7 |
| Mishra AK | 2018 | Low-risk | Low-risk | Low-risk | Low-risk | High-risk | High-risk | High-risk | Unclear | Unclear | Unclear | High-risk | 4 |
| Modi M | 2017 | Low-risk | Low-risk | Low-risk | Low-risk | Low-risk | Low-risk | Unclear | Unclear | Unclear | Unclear | Unclear | 6 |
| Synmon B | 2017 | Low-risk | Low-risk | Low-risk | High-risk | Low-risk | High-risk | Unclear | Unclear | Unclear | Unclear | Unclear | 4 |
| Vibha D | 2010 | Low-risk | Low-risk | Low-risk | Low-risk | Low-risk | Low-risk | Low-risk | Unclear | Unclear | Unclear | Low-risk | 8 |
| Lee TH | 2007 | Low-risk | Low-risk | Low-risk | Low-risk | Low-risk | Unclear | Low-risk | Unclear | Unclear | Unclear | Unclear | 6 |
| Chan KH | 2003 | Low-risk | Low-risk | Low-risk | Low-risk | Low-risk | Unclear | Low-risk | Unclear | Unclear | Unclear | Unclear | 6 |
| Lu CH | 2007 | Low-risk | Low-risk | Low-risk | Low-risk | Unclear | Unclear | Low-risk | Low-risk | Unclear | Unclear | Low-risk | 7 |
| Qu J | 2017 | Low-risk | Low-risk | Low-risk | Unclear | Unclear | Low-risk | Unclear | Unclear | Unclear | Unclear | Low-risk | 5 |
| Singh B | 2012 | Low-risk | Low-risk | Low-risk | Low-risk | Low-risk | Unclear | Low-risk | Low-risk | Low-risk | Low-risk | Low-risk | 10 |
| Khan N | 2017 | Low-risk | Unclear | Unclear | Unclear | Low-risk | High-risk | Low-risk | Unclear | Low-risk | Unclear | Unclear | 4 |
| Siddiqui AA | 2004 | Low-risk | Low-risk | Low-risk | Low-risk | Low-risk | Unclear | Low-risk | Unclear | Unclear | Unclear | Low-risk | 7 |
| Jha SK | 2015 | Low-risk | Low-risk | Low-risk | Low-risk | Low-risk | High-risk | Low-risk | Unclear | Unclear | Low-risk | Unclear | 7 |
| Lo SH | 2019 | Low-risk | Low-risk | Low-risk | Low-risk | Unclear | Unclear | Low-risk | High-risk | High-risk | Unclear | Unclear | 5 |
| de Oliveira L | 2022 | Unclear | Low-risk | Low-risk | Unclear | Low-risk | Low-risk | Low-risk | Unclear | Unclear | Unclear | Unclear | 5 |
| Lee WJ | 2021 | Low-risk | Low-risk | Low-risk | Low-risk | Low-risk | Low-risk | Low-risk | Low-risk | Low-risk | Low-risk | Low-risk | 11 |
| Kamei S | 2005 | Low-risk | Low-risk | Low-risk | Low-risk | Low-risk | Low-risk | Low-risk | Unclear | Unclear | Unclear | Low-risk | 8 |
| Wasay M | 2018 | Low-risk | Low-risk | Low-risk | Unclear | Low-risk | Low-risk | Low-risk | Unclear | Low-risk | Unclear | Unclear | 7 |
| Jacquet P | 2019 | Low-risk | Low-risk | Low-risk | Low-risk | Unclear | Unclear | Low-risk | Unclear | Unclear | Unclear | Low-risk | 6 |
| Cantier M | 2018 | Low-risk | Low-risk | Low-risk | Unclear | Low-risk | Low-risk | Low-risk | Unclear | Unclear | Unclear | Low-risk | 7 |
| Erdem H | 2015 | Low-risk | Low-risk | Low-risk | Low-risk | Low-risk | Low-risk | Low-risk | Unclear | Unclear | Unclear | Low-risk | 8 |
| Sili U | 2014 | Unclear | Low-risk | Low-risk | High-risk | Low-risk | Low-risk | Low-risk | Unclear | Unclear | Unclear | Unclear | 5 |
| Raut T | 2013 | Low-risk | Low-risk | Low-risk | Low-risk | Low-risk | Unclear | Low-risk | Unclear | Low-risk | Low-risk | Unclear | 8 |
| Charlier C | 2018 | Low-risk | Low-risk | Low-risk | Low-risk | Low-risk | Unclear | Low-risk | Unclear | Low-risk | Low-risk | Unclear | 8 |
| Sharma P | 2011 | Low-risk | Low-risk | Low-risk | Low-risk | Low-risk | Low-risk | Low-risk | Unclear | Low-risk | Low-risk | Low-risk | 10 |
| Alam AM | 2022 | Low-risk | Low-risk | Low-risk | Unclear | Unclear | Low-risk | Low-risk | Low-risk | Low-risk | Low-risk | Low-risk | 9 |
| Lu T | 2020 | Low-risk | Low-risk | Low-risk | Low-risk | High-risk | Unclear | Low-risk | Low-risk | Low-risk | Unclear | Unclear | 7 |
| Anuradha HK | 2011 | Low-risk | Low-risk | Low-risk | Low-risk | Low-risk | Low-risk | Low-risk | Low-risk | Unclear | Unclear | Unclear | 8 |
| Lu TT | 2015 | Low-risk | Low-risk | Low-risk | Low-risk | Low-risk | Low-risk | Low-risk | Low-risk | Low-risk | Low-risk | Unclear | 10 |
| Pichler A | 2017 | Low-risk | Low-risk | Low-risk | Low-risk | Low-risk | Unclear | Unclear | Low-risk | Low-risk | Low-risk | Unclear | 8 |
| Marzolf G | 2016 | High-risk | Low-risk | High-risk | Low-risk | Low-risk | Unclear | Low-risk | Low-risk | Unclear | Unclear | Unclear | 5 |
| Zhong Y | 2017 | Low-risk | Low-risk | Low-risk | Low-risk | Unclear | Unclear | Low-risk | Unclear | Unclear | Unclear | Unclear | 5 |
| Bansod A | 2018 | Low-risk | Low-risk | Low-risk | Low-risk | Low-risk | Low-risk | Low-risk | Low-risk | Low-risk | Low-risk | Low-risk | 11 |
| Liu X | 2018 | Low-risk | Low-risk | High-risk | Unclear | Unclear | High-risk | Unclear | Low-risk | Low-risk | Low-risk | Unclear | 5 |
| Kalita J | 2012 | Low-risk | Low-risk | Low-risk | Low-risk | Low-risk | Unclear | Low-risk | Low-risk | Unclear | Unclear | Unclear | 7 |
| Choudhary | 2022 | High-risk | High-risk | High-risk | Low-risk | Unclear | Unclear | Low-risk | Low-risk | Low-risk | Unclear | Low-risk | 5 |
| Choudhary N | 2021 | Unclear | Low-risk | Low-risk | Low-risk | Unclear | Unclear | Low-risk | Low-risk | Low-risk | Unclear | Low-risk | 7 |
| Arsura EL | 2005 | Low-risk | Low-risk | Low-risk | Low-risk | Low-risk | Low-risk | Low-risk | Unclear | Unclear | Unclear | Low-risk | 8 |
| Mirouse A | 2022 | Low-risk | Low-risk | Low-risk | Low-risk | Low-risk | Low-risk | Low-risk | Unclear | Unclear | Unclear | Unclear | 7 |
| Huang H | 2017 | Low-risk | Low-risk | Low-risk | Low-risk | Low-risk | Low-risk | Low-risk | Unclear | Unclear | Unclear | Low-risk | 8 |
| Verma R | 2019 | Low-risk | Low-risk | Low-risk | Low-risk | Low-risk | Low-risk | Low-risk | Unclear | Unclear | Unclear | Unclear | 7 |
| Muralidharan R | 2014 | Low-risk | Low-risk | Low-risk | Low-risk | Unclear | Unclear | Low-risk | Unclear | Unclear | Unclear | Unclear | 5 |
| Kastenbauer S | 2003 | Low-risk | Low-risk | Low-risk | Low-risk | Low-risk | Low-risk | Low-risk | Unclear | Unclear | Unclear | Unclear | 7 |
| Singh TD | 2015 | Low-risk | Low-risk | Low-risk | Low-risk | Low-risk | Low-risk | Low-risk | Unclear | Low-risk | Low-risk | Unclear | 9 |
| Tunthanathip T | 2015 | Low-risk | Low-risk | Low-risk | High-risk | Low-risk | Low-risk | Low-risk | Unclear | Unclear | Unclear | Unclear | 6 |
| Chuang MJ | 2010 | Low-risk | Low-risk | Low-risk | Low-risk | Low-risk | Low-risk | Low-risk | Unclear | Unclear | Unclear | Unclear | 7 |
| Hung C | 2014 | Unclear | Low-risk | Low-risk | Low-risk | Unclear | High-risk | Low-risk | Unclear | Unclear | Unclear | Low-risk | 5 |
| Sheu | 2009 | Low-risk | Low-risk | Low-risk | Low-risk | Low-risk | Unclear | Low-risk | Unclear | Unclear | Unclear | Unclear | 6 |
| Taira N | 2009 | Low-risk | Low-risk | Low-risk | Low-risk | Unclear | Unclear | Low-risk | Unclear | Unclear | Unclear | Unclear | 5 |
| Amornpojnimman T | 2018 | Low-risk | Low-risk | Low-risk | Low-risk | Low-risk | Low-risk | Low-risk | Unclear | Unclear | Unclear | Unclear | 7 |
| Kalita J | 2007 | Low-risk | Low-risk | Unclear | Low-risk | High-risk | Unclear | Low-risk | Unclear | Unclear | Unclear | Unclear | 4 |
| Yasar K | 2010 | Low-risk | Low-risk | Low-risk | Low-risk | Low-risk | Low-risk | Low-risk | Unclear | Unclear | Unclear | Low-risk | 8 |
| Singh T | 2016 | Low-risk | Low-risk | Low-risk | Low-risk | Low-risk | Low-risk | Low-risk | Unclear | High-risk | Low-risk | Unclear | 8 |
| Anuradha HK | 2010 | Low-risk | Low-risk | Low-risk | Low-risk | Low-risk | Low-risk | Low-risk | Low-risk | Unclear | Unclear | Low-risk | 9 |
| Lenhard T | 2016 | Low-risk | Low-risk | Unclear | Low-risk | Low-risk | Low-risk | Low-risk | Low-risk | Unclear | Unclear | Low-risk | 8 |
| Feng B | 2021 | Low-risk | Low-risk | Low-risk | Low-risk | Low-risk | Low-risk | Low-risk | Unclear | Unclear | Unclear | Unclear | 7 |
| Hsu P | 2010 | Unclear | Low-risk | Low-risk | Low-risk | Unclear | Low-risk | Low-risk | Unclear | Unclear | Unclear | Unclear | 5 |
| Gu J | 2015 | Low-risk | Low-risk | Low-risk | Low-risk | Unclear | Low-risk | Low-risk | Unclear | Unclear | Unclear | Unclear | 6 |
| Wasay M | 2004 | Low-risk | Low-risk | Low-risk | Low-risk | Low-risk | Unclear | Low-risk | Unclear | Unclear | Unclear | Low-risk | 7 |
| Clemente Morgado T | 2013 | Low-risk | Low-risk | Low-risk | Low-risk | Unclear | Low-risk | Low-risk | Unclear | Unclear | Unclear | Low-risk | 7 |
| Kim Y | 2016 | Low-risk | Low-risk | Low-risk | Low-risk | Unclear | Low-risk | Low-risk | Low-risk | Unclear | Unclear | Unclear | 7 |
| Wu X | 2021 | Low-risk | Low-risk | Low-risk | Low-risk | Low-risk | Unclear | High-risk | Low-risk | Low-risk | Low-risk | Unclear | 8 |
| Ko SJ | 2014 | Low-risk | Low-risk | Low-risk | High-risk | Unclear | Low-risk | Low-risk | Unclear | Unclear | Unclear | Unclear | 5 |
| Thwaites GE | 2007 | Low-risk | Low-risk | Low-risk | Unclear | Low-risk | Low-risk | Low-risk | Low-risk | Low-risk | Low-risk | Low-risk | 10 |
| Gupta R | 2015 | Low-risk | Low-risk | Low-risk | Low-risk | Low-risk | High-risk | Low-risk | Unclear | Unclear | Low-risk | Unclear | 7 |
| Kumar A | 2022 | Low-risk | Low-risk | Low-risk | Low-risk | Low-risk | Low-risk | Low-risk | Low-risk | Unclear | Unclear | Unclear | 8 |
| Deliran SS | 2022 | Low-risk | Low-risk | Low-risk | Low-risk | Low-risk | Unclear | Low-risk | Unclear | Low-risk | Unclear | Low-risk | 8 |
| Landriel | 2012 | Low-risk | Low-risk | Low-risk | Low-risk | Unclear | Unclear | Low-risk | Unclear | Unclear | Unclear | Unclear | 5 |
| Tsai WC | 2018 | Low-risk | Low-risk | Low-risk | Low-risk | Low-risk | Low-risk | Low-risk | Low-risk | Unclear | Unclear | Low-risk | 9 |
| Singh TD | 2015 | Low-risk | Low-risk | Low-risk | Low-risk | Low-risk | Unclear | Low-risk | Unclear | Unclear | Unclear | Unclear | 6 |
| Sütlaş PN | 2003 | Unclear | Low-risk | Low-risk | Low-risk | Unclear | Unclear | Low-risk | Unclear | Unclear | Unclear | Unclear | 4 |
| Lee J | 2014 | Low-risk | Low-risk | Low-risk | Low-risk | Low-risk | High-risk | High-risk | Low-risk | Low-risk | Low-risk | Unclear | 8 |
| Sinha MK | 2010 | Low-risk | Low-risk | Low-risk | Low-risk | Low-risk | Unclear | Low-risk | Unclear | Low-risk | Low-risk | Unclear | 8 |
| Bhoi SK | 2014 | Low-risk | Low-risk | Low-risk | Unclear | Unclear | Unclear | Low-risk | Unclear | Unclear | Unclear | Unclear | 4 |

Table S5. Descriptive Analysis of studies. Abbreviations : NA Non Available, OR Odds ratio, RR Relative Risk, HR Hazard Ratio, CI confidence interval, PVS PeriVascular Space, mRS modified Rankin Scale, GOS Glasgow Outcome Scale, GOSE, Glasgow Outcome Scale Extended, ASCI Acute or Subacute Cerebral Ischemia, MR / MRI Magnetic Resonance Imaging, CT Computed Tomography, HSE Herpes Simplex Encephalitis, CMV Cytomegalovirus, MBI Modified Barthel Index. We chose to exclude the publication by Sarton et al. from the meta-analysis due to an overlap of patients with the study by Jaquet et al.

| Authors (first) | Year | Design | Centers (n) | Country | Collection period | Number of subjects | Age | CNS infection type | CNS infection type (detailled) | Chosen definition of unfavorable outcome | Unfavorable outcome (n) | Number of CTs | Number of MRIs | Factor set | Association (if not precised, OR are given with 95%CI) |
| --- | --- | --- | --- | --- | --- | --- | --- | --- | --- | --- | --- | --- | --- | --- | --- |
| Kastenbauer S | 2003 | retrospective non-interventional cohort study | 1 | Germany | 1/1984 - 04/2002 | 87 | 50 | Acute Bacterial Meningitis | Pneumococcal Meningitis | Glasgow Outcome Scale ≤ 4 | 45 | NA | NA | Cerebral arterial complication  Cerebral venous complication  Brain swelling | 12.14 (2.6 - 56.78), p<0.001 8.86 (1.06 - 74.25), p = 0.019 8.67 (2.32 - 32.36), p = 0.04 |
| Chan KH | 2003 | retrospective non-interventional cohort study | 1 | China | 01/1997 - 09/2001 | 31 | 43.1 | Acute Mycobacterial Meningitis | Tuberculous Meningitis | Modified Barthel Index ≤12 | 4 | 31 | NA | Hydrocephalus | 36.82 (1.71 - 790.66), p = 0.001 |
| Siddiqui AA | 2004 | retrospective non-interventional cohort study | 2 | Pakistan | 01/1991 - 06/2003 | 25 | 36,5 | Acute Fungal Meningitis | Craniocerebral Aspergillosis of sinonasal origin | Glasgow Outcome Scale ≤ 4 Death | GOS ≤ 4 : 13 Death : 7 | 25 | 20 | Sinonasal disease with only orbital and/or cranial base bony invasion/destruction | 0/7 death (p<0.001) |
| Wasay M | 2004 | retrospective non-interventional cohort study | 1 | Pakistan | 1988 - 1999 | 102 | 30 | Acute Mycobacterial Meningitis | Tuberculoma | Death | 11 | 62 | 30 | Size, location and number of tuberculoma  Hydrocephalus  Concomittant tuberculous meningitis | 1.2 (0.6 - 2.2), p = 0.2 1.3 (0.8 - 2.2), p = 0.12 1.8 (0.8 - 2.6), p = 0.08 |
| Arsura EL | 2005 | retrospective non-interventional cohort study | 1 | USA | 1991 - 1997 | 63 | 35 | Acute Fungal Meningitis | Coccidioidal Meningitis | Death | 14 | 36 | 50 | Hydrocephalus  Hydrocephalus and cerebral infarction | 12.5-fold increased mortality rate, p < 0.03 11.8-fold increased mortality rate, p < 0.02 |
| Xiao F | 2005 | retrospective non-interventional cohort study | 1 | Taiwan | 1986 - 2002 | 178 | 43 | Brain Abscess | Miscellaneous Brain Abscess | Severe neurological sequellae or death | 67 | NA | NA | Deep-seated location  Supratentorial (not deep-seated)  Intraventricular rupture | 3.356 (1.056 - 10.664), p = 0.032 0.818 (0.665 - 1.006), p = 0.056 1.678 (0.243 - 11.598), p = 0.63 |
| Kamei S | 2005 | retrospective non-interventional cohort study | 6 | Japan | 1996 | 45 | 46 | Viral Encephalitis | Herpes Simplex Encephalitis | Death or neurological sequellae | 19 | 45 | 45 | Abnormal head CT  Abnormal head MR | 6.22 (1.45 - 26.65), p = 0.235 5.4 (0.59 - 49.27), p = 0.135 |
| Lu CH | 2006 | prospective non-interventional cohort study | 1 | Taiwan | 07/2004 - 07/2005 | 24 | 49,6 | Acute Bacterial Meningitis | NA | Modified Barthel Index ≤12 | 8 | NA | 24 | Hydrocephalus  Cerebral infarction  Leptomeningeal enhancement  Intracranial stenoses | 0.076 (0.16 - 0.57), p = 0.33 0.20 (0.02 - 2.64), p = 0.249 0.24 (0.03 - 1.87), p = 0.29 0.20 (0.02 - 2.64), p = 0.249 |
| Tseng JH | 2006 | retrospective non-interventional cohort study | 1 | Taiwan | 01/1986 - 12/2004 | 142 | 41,5 | Brain Abscess | Miscellaneous Brain Abscess | Glasgow Outcome Scale ≤ 3 | 37 | 142 | NA | Location  Multiple abscesses | No statistical association with outcome, p = NA  No statistical association with outcome, p = NA |
| Kalita J | 2007 | prospective non-interventional cohort study | 1 | India | NA | 90 | 33.2 | Acute Mycobacterial Meningitis | Tuberculous Meningitis | neurological sequellae | 51 | 90 | NA | Optic atrophy | Significatively associated with neurological sequellae, p = 0.001 |
| Thwaites GE | 2007 | randomized controlled interventional study | 2 | Vietnam | 04/2001 - 04/2003 | 43 | 31* | Acute Mycobacterial Meningitis | Tuberculous Meningitis | modified Rankin Scale ≥3 | 29 | NA | 83 | MRI appearance at diagnosis | No statistical association with outcome |
| Demir MK | 2007 | retrospective non-interventional cohort study | 1 | Turkey | NA | 96 | 29,7* | Brain Abscess | Bacterial Brain Abscess | Any adverse event | 41 | 96 | 41 | ISI score ≥ 9 | Associated to outcome (p < 0.001) |
| Lee TH | 2007 | retrospective non-interventional cohort study | 1 | Taiwan | 1986 - 2005 | 179 | 47 | Brain Abscess | Miscellaneous Brain Abscess | Glasgow Outcome Scale ≤ 4* Death | GOS ≤ 4 : 84 Death : NA | NA | NA | Intraventricular rupture (death)  Intraventricular rupture (GOS) | 1.11 (0.36 - 3.39), p = 1 No statistical association with outcome, p = 0.276 |
| Sheu | 2009 | retrospective non-interventional cohort study | 1 | Taiwan | 01/1997 - 12/2006 | 117 | NA | Acute Mycobacterial Meningitis | Tuberculous Meningitis | Severe sequelae or death | 28 | 117 | NA | Hypodense lesions on the brain image | 3.81 (1.52 - 9.60), p = 0.004 |
| Taira N | 2009 | retrospective non-interventional cohort study | 1 | Japan | 1996 - 2007 | 23 | 46,4 | Viral Encephalitis | Herpes Simplex Encephalitis | prolonged course | 8 | 23 | NA | abnormal lesions on cranial CT  Detection of lesions on initial CT | 87.5% (prolonged course) 33.3%(non-prolonged course), p = 0.027 100.0% (prolonged course), 66.7% (non-prolonged course), p = 0.112 |
| Vibha D | 2010 | retrospective non-interventional cohort study | 1 | India | 07/2004 - 09/2008 | 380 | NA | Acute Bacterial Meningitis | NA | Death | 34 | 224 | NA | Exudates  Brain edema  Ischemia  Hydrocephalus | 195 (48.42 - 785.26), p < 0.001 38.88 (2.33 - 648.72), p < 0.001 6.47 (1.68 - 24.87), p = 0.002 1.42 (0.3 - 6.76), p = 0.655 |
| Anuradha HK | 2010 | prospective non-interventional cohort study | 1 | India | 03/2008 - 03/2009 | 100 | 30 | Acute Mycobacterial Meningitis | Tuberculous Meningitis | Ischemia on MRI modified Rankin Scale ≥3 | Ischemia : 27 mRS ≥3 : 27 | NA | 100 | Basal exudates (ischemia)  Sylvian fissure exudates (ischemia)  Optochiasmatic exudates (ischemia)  MCA ischemia (mRS ≥3) | 1.3 (1.01 - 1.67), p = 0.042 1.29 (1.00 - 1.67), p = 0.026 1.51 (0.98 - 2.34), p = 0.02 1.33 (0.91 - 1.95), p = 0.001 |
| Sinha MK | 2010 | prospective non-interventional cohort study | 1 | India | 04/2008 - 08/2009 | 101 | 30 | Acute Mycobacterial Meningitis | Tuberculous Meningitis | Death or disability | 27 | NA | 101 | Ischemia | 1.28 (0.99-1.76), p = 0.038 |
| Yasar K | 2010 | retrospective non-interventional cohort study | 1 | Turkey | 01/1998 - 03/2009 | 160 | 32,18 | Acute Mycobacterial Meningitis | Tuberculous Meningitis | Modified Barthel Index ≤12 | 33 | 160 | 136 | Neuroradiological signs on CT/MRI | 0.498 (0.08 - 3.07), p = 0453 |
| Hsu P | 2010 | retrospective non-interventional cohort study | 1 | Taiwan | 01/2000 - 09/2006 | 108 | 54,9 | Acute Mycobacterial Meningitis | Tuberculous Meningitis | Death | 42 | NA | NA | Hydrocephalus  Ischemia | 4.977 (1.345 - 18.410), p = 0.016 1.77 (0.69 - 4.56), p = 0.392 |
| Chuang MJ | 2010 | retrospective non-interventional cohort study | 1 | Taiwan | 1986 - 2007 | 205 | 46,2 | Brain Abscess | NA | Seizures (early or late onset) | 48 | 202 | NA | Fronto-parietal distribution of bacterial brain abscesses  Hydrocephalus | 2.58 (1.11 - 6.03), p = 0.028 1.22 (.53 - 2.83), p =.645 |
| Sharma P | 2011 | retrospective non-interventional cohort study | 1 | India | 01/2008 - 03/2010 | 158 | 31,95 | Acute Mycobacterial Meningitis | Tuberculous Meningitis | modified Rankin Scale ≥3 | 60 | NA | 158 | No statistical correlation |  |
| Anuradha HK | 2011 | retrospective non-interventional cohort study | 1 | India | NA | 110 | 31 | Acute Mycobacterial Meningitis | Tuberculomas | modified Rankin Scale ≥3 | 32 | NA | 110 | Tuberculoma  Meningeal enhancement  Hydrocephalus | 2.27 (0.98 - 5.24), p > 0.05 0.85 (0.655 - 2.363), p = 0.529 no statistical association with outcome, p = NA |
| Nathoo N | 2011 | retrospective non-interventional cohort study | 1 | South Africa | 1983 - 2002 | 973 | 24,36 | Brain Abscess | Miscellaneous Brain Abscess | Death | Death : 130 | NA | NA | Cerebral infarction  Ventriculitis  Hydrocephalus  Multiple abscesses | 31.1 (NA), p < 0.05 (but NA) 12.4 (6.37 - 23.14), p = 0.00001 5.1 (NA), p < 0.001 3.1 (1.68 - 6.69), p = 0.0001 |
| Kalita J | 2012 | retrospective non-interventional cohort study | 1 | India | NA | 67 | 34* | Acute Mycobacterial Meningitis | Tuberculous Meningitis | Modified Barthel Index ≤12 | 25 | NA | 67 | Baseline MRA abnormality | No statistical association (p = 0.63) |
| Singh B | 2012 | prospective non-interventional cohort study | 1 | India | 11/2009 - 10/2011 | 47 | 28 | Acute Mycobacterial Meningitis | Tuberculous Meningitis | Modified Barthel Index ≤12 | 12 | 47 | NA | Hydrocephalous  Basal exudates  Ischemia | Significatively associated with poorer outcome, p = 0.026 Significatively associated with poorer outcome, p = 0.013 Significatively associated with poorer outcome, p = 0.003 |
| Landriel | 2012 | retrospective non-interventional cohort study | 1 | Argentina | 02/2001 - 01/2010 | 59 | 33,69 | Brain Abscess | Miscellaneous Brain Abscess | Glasgow Outcome Scale ≤ 4 | 11 | NA | NA | Multiple abscesses  Superficial location | 1.62 (0.36 - 7.37), p = 0.679 0.38 (0.08 - 1.84), p = 0.16 |
| Tan IL | 2012 | retrospective non-interventional cohort study | 1 | USA | 01/1997 - 04/2010 | 29 | 55,1 | Viral Encephalitis | Herpes Simplex Encephalitis | Karnofsky Performance Status Scale (linear evaluation) | NA | NA | 28 | Bitemporal lesions on MRI | No statistical association with KPSS |
| Raut T | 2013 | prospective non-interventional cohort study | 1 | India | 10/2010 - 08/2012 | 80 | 30.1 | Acute Mycobacterial Meningitis | Tuberculous Meningitis | Modified Barthel Index ≤12 | 23 | NA | 80 | Hydrocephalus  Basal exudate | 8.81 (1.89 - 41.13), p = 0.02 8.81 (1.89 - 41.13), p = 0.001 |
| Clemente Morgado T | 2013 | retrospective non-interventional cohort study | 1 | South Africa | 2005 - 2011 | 22 | 31 | Acute Mycobacterial Meningitis | Tuberculous Meningitis With Hydrocephalus | Death | 15 | 10 | NA | Ischemia | HR 95%CI 5.4 (1.6 - 18.5), p < 0.05 |
| Hong D | 2013 | retrospective non-interventional cohort study | 1 | China | 2000 - 2010 | 26 | 23,27 | Parasitic encephalitis | Cerebral Sparganosis | Seizure relapse or hemiparesis | 9 | NA | 26 | Tunnel sign  Lesion migration on 2 different MRIs | 1.91 (1.22–3.14), p = 0.002 2.43 (1.37–4.53), p = 0.003 |
| Muralidharan R | 2014 | retrospective non-interventional cohort study | 1 | USA | 01/00 - 11/10 | 39 | 54* | Acute Bacterial Meningitis | Fulminant Bacterial Meningitis | modified Rankin Scale ≥2 | 31 | 36 | NA | Abnormal head imaging at presentation | 9,4 (1,78 - 49,6), p = 0.008 |
| Hung C | 2014 | retrospective non-interventional cohort study | 1 | Taiwan | 1986 - 2007 | 180 | 52.3 | Acute Fungal Meningitis | Cryptococcal Meningitis | Seizures Death | Seizures : 28 Death : 180 | NA | NA | Hydrocephalus (seizures)  Ischemia (seizures)  Basal cistern effacement (seizures)  Hydrocephalus (death)  Ischemia (death)  Basal cistern effacement (death) | 1.405 (0.623 - 3.170), p = 0.411 1.643 (0.498 - 5.415), p = 0.489 0.526 (0.065 - 4.279), p = 1 1.293 (0.689 - 2.428), p = 0.423 1.088 (0.387 - 3.062), p = 0.873 2.776 (0.81 - 9.513), p = 0.092 |
| Lee J | 2014 | retrospective non-interventional cohort study | 1 | Korea | 11/2010 - 02/2013 | 43 | 37,65 | Acute Infectious Meningitis (Multiple Agents) | Aseptic 18 (41.9) Bacteria 8 (18.6) Virus 9 (20.9) Fungus 3 (7) Tuberculosis 4 (9.3) Parasite 1 (2.3) | Neurological sequellae | 4 | NA | 43 | Leptomeningeal enchancement score | Recovery group 3.26 ± 4.29 ; Complicated group 10.25 ± 2.75, p = 0.007 |
| Wasay M | 2014 | retrospective non-interventional cohort study | 1 | Pakistan | 2002 - 2011 | 404 | 43 | Acute Mycobacterial Meningitis | Tuberculous Meningitis | modified Rankin Scale ≥4 | 155 | 153 | 313 | Hydrocephalus  Infarction  Tuberculuoma | 1.95 (NA - NA), p = NA 5.50 (3.36 - 8.99), p = NA 0.79 (0.53 - 1.18), p = NA |
| Bhoi SK | 2014 | retrospective non-interventional cohort study | 1 | India | 2003 - 2013 | 21 | 30* | Viral Encephalitis | Dengue Meningitis | Death or disability | 2 | NA | NA | Abnormal imaging | no statistical association with outcome, p = 0.16 |
| Ko SJ | 2014 | retrospective non-interventional cohort study | 1 | Korea | 01/2004 - 12/2014 | 51 | 53 | Brain Abscess | Miscellaneous Brain Abscess | Glasgow Outcome Scale ≤ 3 | 10 | NA | NA | Multiple abscesses | 1.80 (0.29 - 11.00), p = 0.612 |
| Sili U | 2014 | retrospective non-interventional cohort study | 17 | Turkey | NA | 101 | 44 | Viral Encephalitis | Herpes Simplex Encephalitis | Morbidity (Whitley et al) | 26 | NA | 98 | Bilateral involvement  Extensive involvement | 2.05 (0.41 - 10.34), p = 0.39 37.22 (7.09 - 195.47), p < 0.001 |
| Sporrborn JL | 2015 | retrospective non-interventional cohort study | 2 | Denmark | 2003 - 2010 | 107 | 64* | Acute Bacterial Meningitis |  | Mortality at 30-days | 26 | 81 | NA | Hydrocephalus (defined as highest ventricule/brain ratio tertile) | 8.78 (1.74 - 44.36), p = 0.009 |
| Zheng H | 2015 | retrospective non-interventional cohort study | 1 | China | 1998 - 2013 | 108 | 37,4 | Acute Fungal Meningitis | Cryptococcal Meningitis | modified Rankin Scale ≥3 | 63 | NA | 48 | Abnormal MRI | 10 death for 34 abnormal MRIs, p>0.05 |
| Jha SK | 2015 | prospective non-interventional cohort study | 1 | India | 10/2012 - 01/2014 | 118 | NA | Acute Mycobacterial Meningitis | Tuberculous Meningitis | Modified Barthel Index ≤12 | 26 | NA | 118 | Ischemia  Hydrocephalus | 0.410 (0.053 - 3.180), p = NA 0.360 (0.097 - 1.332), p = NA |
| Erdem H | 2015 | retrospective non-interventional cohort study | 43 | Turkey | 2000 - 2012 | 507 | NA | Acute Mycobacterial Meningitis | Tuberculous Meningitis | Death or neurological sequellae | 165 | NA | NA | Hydrocephalus  Abscess  Basal exudate  Tuberculoma  Vasculitis | 2.02 (1.32 - 3.08), p < 0.01 2.57 (1.13 - 5.88), p = 0.039 1.61 (1.04 - 2.49), p = NA 1.41 (0.92 - 2.18), p = NA 2.29 (1.45 - 3.63), p < 0.01 |
| Lu T | 2015 | prospective non-interventional cohort study | 1 | China | 01/2007 - 07/2014 | 101 | 36,7 | Acute Mycobacterial Meningitis | Tuberculous Meningitis | Modified Barthel Index ≤12 | 9 | NA | 101 | Abnormal Magnetic Resonnance Angiogrophy  Tuberculoma  Hydrocephalus | No statistical association with outcome, p = NA No statistical association with outcome, p = NA No statistical association with outcome, p = NA |
| Gu J | 2015 | retrospective non-interventional cohort study | 1 | China | 01/2008 - 09/2012 | 156 | 32,9 | Acute Mycobacterial Meningitis | Tuberculous Meningitis | Death or neurological sequellae | 60 | NA | NA | Hydrocephalus  Ischemia | 5.031 (1.389 - 13.347), p = 0.023 1.82 (0.83 - 3.98), p = 0.316 |
| Gupta R | 2015 | prospective non-interventional cohort study | 1 | India | 11/2011 - 11/2013 | 71 | 30,2 | Acute Mycobacterial Meningitis | Tuberculous Meningitis | Modified Barthel Index ≤12 | 28 | NA | 71 | Basal exudates  Spinal meningeal enhancement | RR 95%CI 5.506 (1.689 - 17.949), p = 0.003 RR 95%CI 3.635 (1.057 - 12.495), p = 0.034 |
| Tunthanathip T | 2015 | retrospective non-interventional cohort study | 1 | Thailand | 01/1999 - 12/2013 | 114 | 41* | Brain Abscess | Miscellaneous Brain Abscess | Glasgow Outcome Scale Extended ≤ 4 | 43 | NA | NA | Intraventricular rupture | 5.50 (1.34 - 22.49), p = 0,017 |
| Marzolf G | 2016 | retrospective non-interventional cohort study | 7 | France | 2006 - 2013 | 21 | NA | Acute Fungal Meningitis | CNS Aspergillosis | mortality within 12 weeks | 8 | NA | 21 | Hematogenous spread | Higher 12-week mortality rate than patients with direct spread, p = 0.046 |
| Lenhard T | 2016 | prospective non-interventional cohort study | 2 | Germany | 04/2004 - 09/2014 | 111 | 51* | Viral Encephalitis | Tick-Borne Encephalitis | modified Rankin Scale (continuous scale) | NA | NA | NA | Associated Radicular Lesion | Significatively associated with poorer outcome (p<0.0001) |
| Singh T | 2016 | retrospective non-interventional cohort study | 1 | USA | 01/1995 - 12/2013 | 45 | 66* | Viral Encephalitis | Herpes Simplex Encephalitis | modified Rankin Scale ≥3 | 14 | 42 | 40 | Restricted diffusion | 8.54 (1.87 - 51.74), p = 0.005 |
| Kim Y | 2016 | retrospective non-interventional cohort study | 1 | Korea | 2000 - 2004 | 29 | 42,21* | Viral Encephalitis | Herpes Simplex Encephalitis | Glasgow Outcome Scale ≤ 3* | 15 | NA | 25 | Abnormal head MR  Restricted diffusion  Lesional volume on FLAIR | No statistical association with outcome, p = NA 0.533 (0.1 - 2.839), p = 0.461 0.996 (0.970 - 1.022), p = 0.757 |
| Khan N | 2017 | prospective non-interventional cohort study | 1 | South Africa | 01/2012 - 12/2013 | 30 | NA | Acute Fungal Meningitis | Cryptococcal Meningitis | Death | 10 | 30 | NA | Changes on imaging  Hydrocephalus | No statistical association with outcome 41 (1.95 - 860.78), p = NA |
| Zhong Y | 2017 | retrospective non-interventional cohort study | 1 | China | 2002 - 2014 | 114 | NA | Acute Fungal Meningitis | Cryptococcal Meningitis | Glasgow Outcome Scale ≤ 3 | 40 | NA | 114 | Abnormal MRI | 2.67 (1.06 - 6.7), p = 0.033 |
| Qu J | 2017 | retrospective non-interventional cohort study | 1 | China | 01/2008 - 12/2015 | 231 | NA | Acute Infectious Meningitis (Multiple Agents) | Cryptococcus Meningitis Tuberculous Meningitis | NA | 70 | NA | 187 | Hydrocephalus (tuberculous meningitis)  Demyelination (Cryptococcus meningitis) | 7.290 (1.630 - 32.606), p = 0.009 7.270 (2.560 - 20.646), p < 0.001 |
| Modi M | 2017 | prospective non-interventional cohort study | 1 | India | 01/2011 - 01/2015 | 209 | 30.4 | Acute Mycobacterial Meningitis | Tuberculous Meningitis | Death | 53 | NA | 209 | Hydrocephalus (death) | 3.2 (1.5 - 6.7), p = 0.003 |
| Synmon B | 2017 | prospective non-interventional cohort study | 1 | India | 08/2013 - 05/2015 | 93 | 32.3 | Acute Mycobacterial Meningitis | Tuberculous Meningitis | Death or disability | 63 | 93 | 47 | Hydrocephalus (death or disability)  Hydrocephalus (death only)  Vasculitis (death or disability) | 1.06 (0.33 - 3.37), p = NA 4.10 (1.30 - 12.93), p = NA 3.25 (1.00 - 10.53), p = NA |
| Huang H | 2017 | retrospective non-interventional cohort study | 1 | China | 01/2013 - 01/2015 | 45 | 46,04 | Acute Mycobacterial Meningitis | Tuberculous Meningitis | Glasgow Outcome Scale ≤ 4 | 10 | NA | NA | Hydrocephalus | 11.00 (1.63 - 74.08) |
| Pichler A | 2017 | retrospective non-interventional cohort study | 1 | Austria | 2003 - 2014 | 45 | 58* | Viral Encephalitis | Tick-Borne Encephalitis | Neurological sequellae | 28 | NA | 45 | Parenchymal lesions | No statistical association with outcome, p = NA |
| Charlier C | 2018 | retrospective non-interventional cohort study | NA | France | 11/2009 - 10/2013 | 71 | 64* | Acute Bacterial Meningitis | Neurolisteriosis | Mortality at 30-days | 23 | NA | NA | Parenchymal lesions (abscess(es), nodule(s) and/ or non-specific white matter lesions  Hydrocephalus or contrast-enhancing ventricles | 5.60 (1.42 - 29.6), p = 0.02 5,96 (0.73 - 130.17), p = 0.14 |
| Mishra AK | 2018 | retrospective non-interventional cohort study | 1 | India | 05/2004 - 08/2015 | 66 | 41 | Acute Fungal Meningitis | Cryptococcal Meningitis | Death Acute or Subacute Cerebral Ischemia (ASCI) | Death : 30 ASCI : 20 | 66 | 22 | Basal exudates (ASCI)  Hydrocephalus (ASCI)  Acute or Subacute Cerebral Ischemia (death) | 18.6 (1.92 - 179.73), p = 0.022 0.9 (0.25 - 3.3), p = 1 9.14 (2.59 - 32.32), p < 0.0001 |
| Tsai WC | 2018 | prospective non-interventional cohort study | 1 | Taiwan | 01/2001 - 12/2015 | 65 | 59* | Acute Fungal Meningitis | Cryptococcal Meningitis In HIV-Negative Patients | Glasgow Outcome Scale ≤ 3 Acute or Subacute Cerebral Ischemia (ASCI) | GOS ≤ 3 : 28 ASCI : 14 | NA | 65 | Acute or Subacute Cerebral Ischemia (ASCI) (GOS)  Hydrocephalus (GOS)  Basal meningeal enchancement (GOS)  Basal meningeal enchancement (ASCI)  Hydrocephalus (ASCI) | 13.13 (2.63 - 65.66), p < 0.001 1.75 (0.61 - 5), p = 0.295 1.47 (0.55 - 3.95), p = 0.256 2.79 (0.82 - 9.54), p = 0.042 1.8 (0.53 - 6.08), p = 0.353 |
| Bansod A | 2018 | prospective non-interventional cohort study | 1 | India | NA | 107 | 29,8 | Acute Mycobacterial Meningitis | Tuberculous Meningitis | Modified Barthel Index ≤12 or death at 6 months | 24 | NA | 107 | Abnormal Magnetic Resonnance Veinography | 2.86 (0.82 - 10.01) |
| Wasay M | 2018 | retrospective non-interventional cohort study | 1 | Pakistan | 2002 - 2013 | 559 | 47.8 | Acute Mycobacterial Meningitis | Tuberculous Meningitis | Ischemia Death | Ischemia : 144 Death : 77 | 209 | 452 | Ischemia (death) | 3.84 (2.27 - 6.49), p = NA |
| Cantier M | 2018 | retrospective non-interventional cohort study | 12 | France | 2004 - 2016 | 90 | 43* | Acute Mycobacterial Meningitis | Tuberculous Meningitis | modified Rankin Scale ≥3 | 55 | 90 | 75 | Hydrocephalus | 4.79 (1.48 - 15.50), p = NA |
| Amornpojnimman T | 2018 | retrospective non-interventional cohort study | 1 | Thailand | 2002 - 2017 | 81 | 47,68 | Brain Abscess | Miscellaneous Brain Abscess | death | 11 | NA | NA | multi-loculated abscess | Significatively associated with mortality, p = 0.011 |
| Liu X | 2018 | retrospective non-interventional cohort study | 1 | China | NA | 30 | 14,3* | Viral Encephalitis | Miscellaneous Viral infection (CMV, HSE) | Group I : Glasgow Coma Scale (GCS) ≥13 and recovered with no sequelae; Group II: GCS 9–12 and recovered with some sequelae; Group III: GCS 3–8 and recovered with more severe sequelae | Group 1 : 12 Groupe II : 11 Group III : 7 | NA | 30 | SWI MinIP volume (Cc) | Group I 0.08 +/- 0.27 ; group II 0.87 +/- 0.81 ; group III 3,06 +/- 0.23, p < 0.001 |
| Zhang L | 2019 | prospective non-interventional cohort study | 1 | China | 02/2014 - 12/2017 | 52 | 30.3 | Acute Mycobacterial Meningitis | Tuberculous Meningitis | modified Rankin Scale ≥3 Acute or Subacute Cerebral Ischemia (ASCI) | mRS ≥3 : 11 ASCI : 12 | NA | 52 | Acute or Subacute Cerebral Ischemia (mRS)  Basal exudates (ASCI)  Tuberculoma (ASCI) | 4.50 (0.92 - 21.92), p = 0.024 23.913 (1.398 - 408.975), p = NA 0.005 (0.000 - 0.254), p = NA |
| Verma R | 2019 | prospective non-interventional cohort study | 1 | India | 10/2015 - 03/2017 | 101 | 32,49 | Acute Mycobacterial Meningitis | Tuberculous Meningitis | Modified Barthel Index ≤12 | 33 | NA | 101 | Hydrocephalus  Basal exudate  Ischemia  Tuberculoma  Optochiasmatic arachnoiditis | 6.548 (1.667 - 25.718), p = 0.003 1.845 (0.544 - 6.263), p = 0.321 1.964 (0.466 - 8.270), p = 0.397 1.733 (0.512 - 5.864), p = 0.373 3.850 (0.830 - 17.861), p = 0.101 |
| Lo SH | 2019 | retrospective non-interventional cohort study | 5 | Taiwan | 2001 - 2018 | 68 | 50* | Viral Encephalitis | Japanese Encephalitis | GCS at discharge ≤ 8 | 16 | 61 | 61 | Thalamus involvement  Hippocampus involvement  Midbrain involvement  Pons involvement  Basal Ganglion involvement  Meningeal enhancement | 2.396 (0.658-8.229), p = 0.185 2.286 (0.558 - 9.366), p = 0.251 4.300 (0.915 - 20.205), p = 0.065 1.128 (0.108 - 11.785), p = 0.92 1.180 (0.382 - 3.42), p = 0.774 1.480 (0.382 - 5.730), p = 0.57 |
| Jacquet P | 2019 | retrospective non-interventional cohort study | 47 | France | 2007 - 2017 | 259 | 64* | Viral Encephalitis | Herpes Simplex Encephalitis | modified Rankin Scale ≥3 | 185 | 214 | 223 | Involvement > 3 lobes  Abnormal head MR  Abnormal head CT | 3.04 (1.35 - 6.81), p <0.01 7.27 (0.74 - 71.16), p = 0.04 0.88 (0.47 - 1.65), p = 0.69 |
| Lu T | 2020 | retrospective non-interventional cohort study | 1 | China | 11/2015 - 06/2018 | 27 | 40,15 | Acute Mycobacterial Meningitis | Tuberculous Meningitis | Ischemia | 8 | NA | 27 | M1 perivascular enhancement  M1 surrounded by exudates  Wall thickening | 12.05 (1.13 - 128.45), p = 0.024 25.2 (2.77 - 229.57), p < 0.001 10.89 (1.23- 96.09), p = 0.019 |
| Lee WJ | 2021 | retrospective non-interventional cohort study | 1 | Korea | 01/2000 - 12/2019 | 76 | 55,4 | Acute Fungal Meningitis | Cryptococcal Meningitis | modified Rankin Scale ≥3 | 34 | NA | 76 | Total ePVS score  ePVS value score ≥5  Periventricular extension | 4.331 (1.457 - 12.875), p = 0.008 60.073 (5.152 - 700.485), p = 0.001 23.106 (2.796 - 297.176), p = 0.016 |
| Choudhary N | 2021 | prospective non-interventional cohort study | 1 | India | NA | 101 | 30 | Acute Mycobacterial Meningitis | Tuberculous Meningitis | Ischemia on MRI | 49 | NA | 101 | Vessel wall enhancement | 37.6 (8.21 - 172.16), p < 0.001 |
| Feng B | 2021 | retrospective non-interventional cohort study | 1 | China | 01/2008 - 04/2018 | 80 | 38,5 | Acute Mycobacterial Meningitis | Tuberculous Meningitis | Glasgow Outcome Scale ≤ 3 | 39 | 80 | NA | Hydrocephalus  Ischemia  Basal exudate  Tuberculoma | 2.25 (0.85 - 5.94), p = 0.15 1.83 (0.72, 4.64), p = 0.25 1.10 (0.29 - 4.18), p > 0.99 3.47 (0.34 - 35.02), p = 0.34 |
| Wu X | 2021 | retrospective non-interventional cohort study | 1 | China | 05/2019 - 12/2020 | 60 | 33,37 | Acute Mycobacterial Meningitis | Tuberculous Meningitis | Modified Barthel Index ≤12 | 35 | NA | 60 | Exudates  Hydrocephalus  Ischemia | 0.301 (0.026-2,768), p = 0.3 0.057 (0.003-0.444), p = 0.018 0.814([0.080 - 8,77), p = 0.86 |
| Sarton B | 2021 | prospective non-interventional cohort study | 34 | France | 2007 - 2019 | 138 | 62,6* | Viral Encephalitis | Herpes Simplex Encephalitis | modified Rankin Scale ≥3 | 95 | NA | 138 | Involvement > 3 lobes  Extensive bilateral involvement  Focal diffusion hyperintensity  Bilateral diffusion abnormalities  Abnormalities in right thalamus on T2*-sequence | 25.7 (1.21 - 554.42), p = NA 3.17 (0.64 - 17.65), p = NA 6.90 (1.12 - 43), p = NA Significatively associated with poorer outcome, p =0.03 0.21 (0.41 - 1.02), p = 0.05 |
| Deliran SS | 2022 | prospective non-interventional cohort study | NA | Netherlands | 03/2006 - 07/2018 | 24 | 65 | Acute Bacterial Meningitis | NA | Glasgow Outcome Scale ≤ 4 Death | GOS ≤ 4 : 19 Death : 12 | 22 | NA | Subarachnoid Hemorrhage on CT | 6.3 (2.7 - 14.69), p < 0.001 (mortality)  1.87 (3.21 - 36.84), p <.1 (GOS ≤ 4) |
| Tu J | 2022 | retrospective non-interventional cohort study | 1 | China | 01/2016 - 02/2022 | 53 | NA | Acute Fungal Meningitis | Cryptococcal Meningitis | modified Rankin Scale ≥3 Acute or Subacute Cerebral Ischemia (ASCI) | mRS ≥3 : 14 ASCI : 14 | NA | 53 | Ischemia (mRS)  Hydrocephalus (ASCI)  Posterior fossa exudates (ASCI) | 12.24 (2.9 - 51.71), p = 0.001 3.875 (1.051 - 14.282), p = 0.036 3.929 (1.029 - 14.992), p = 0.045 |
| de Oliveira L | 2022 | retrospective non-interventional cohort study | 1 | Brazil | 08/2012 - 08/2017 | 96 | 38.7 | Acute Fungal Meningitis | Cryptococcal Meningitis | Death | 25 | 90 | 90 | Brain edema  Hydrocephalus  Abnormal neuroimaging | 10.58 (1.04 - 107.64), p = 0.04 10.58 (1.04 - 107.64), p = 0.04 41,73 (3,10-561,65), p = 0.005 |
| Choudhary | 2022 | prospective non-interventional cohort study | 1 | India | NA | 12 | NA | Acute Infectious Meningitis (Multiple Agents) | Suspected Bacterial And Fungal Cns Infections | Ischemia on MRI | 12 | NA | 24 | Vessel wall enhancement | Significatively associated with ischemia, p = 0.04 |
| Cao X | 2022 | retrospective non-interventional cohort study | 1 | India | 01/2010 - 01/2019 | 110 | 43.17 | Acute Mycobacterial Meningitis | Tuberculous Meningitis | modified Rankin Scale ≥3 | 7 | NA | 109 | Meningeal enhancement  Tuberculoma  Acute ischemia  Old ischemia  Abscess  Hydrocephalus | 1,387 (0.325 - 5,925), p = 0.658 2,040 (0.520 - 8,000), p = 0.306 6,375 (1,501 - 27,080), p = 0.012 5.556 (1.332 - 23.177), p = 0.019 4,667 (0.673 - 32,360), p = 0.119 9,139 (2,052 - 40.700), p = 0.004 |
| Kumar A | 2022 | prospective non-interventional cohort study | 1 | India | 09/2018 - 09/2020 | 120 | 26* | Acute Mycobacterial Meningitis | Tuberculous Meningitis | Death Acute or Subacute Cerebral Ischemia (ASCI) | Death : 19 ASCI : 46 | NA | 120 | Basal exudates (ischemia)  Ischemia (death)  Hydrocephalus (death)  Basal exudates (death) | 3.00 (1.39 - 6.49), p = 0,018 3.38 (1.22 - 9.36), p = 0,016 No statistical association with outcome, p = NA  No statistical association with outcome, p = 0.05 |
| Mirouse A | 2022 | retrospective non-interventional cohort study | 18 | France | 2000 - 2017 | 55 | 53* | Viral Encephalitis | VZV Meningitis And Meningo-Encephalitis | modified Rankin Scale ≥3 | 30 | 33 | 30 | Vasculitis on brain MRI | 0.44 (0.076 - 2.576), p = 0.35 |
| Sonneville R | 2023 | prospective non-interventional cohort study | 68 | France | 2017 - 2020 | 599 | 66* | Infectious Encephalitis (Multiple agents) | Acute Bacterial Meningitis 247 (41.8) Infectious Encephalitis 140 (23.7) Autoimmune 38 (6.4) Neoplastic/Toxic 11 (1.9) Unknown Origin 155 (26.2) | modified Rankin Scale ≥3 | 298 | 495 | 365 | Abnormal head CT  Abnormal head MR | 2.43 (1.66 - 3.55), p <0.01 2.81 (1.71 - 4.63), p <0.01 |





Figure S1. Forest plot illustrating the relationship between hydrocephalus and mortality across included studies. “Quality index” refers to the quality scale adapted from NOS and RoBANS.





Figure S2. Forest plot illustrating the relationship between brain ischemia and mortality across included studies. “Quality index” refers to the quality scale adapted from NOS and RoBANS. Detailed description for each study is available in supplemental material.


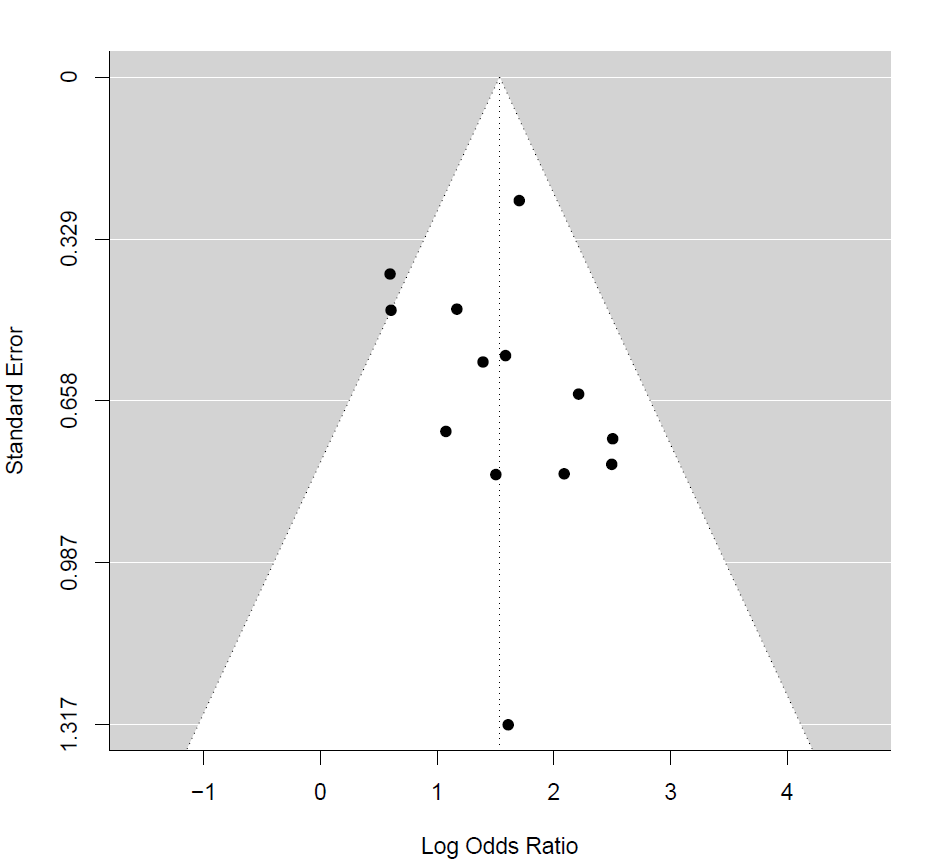


Figure S3. Diagnostic plot evaluating distribution of the 13 studies for association between brain ischemia and functional outcomes (p-value for asymmetrical distribution, 0.516)


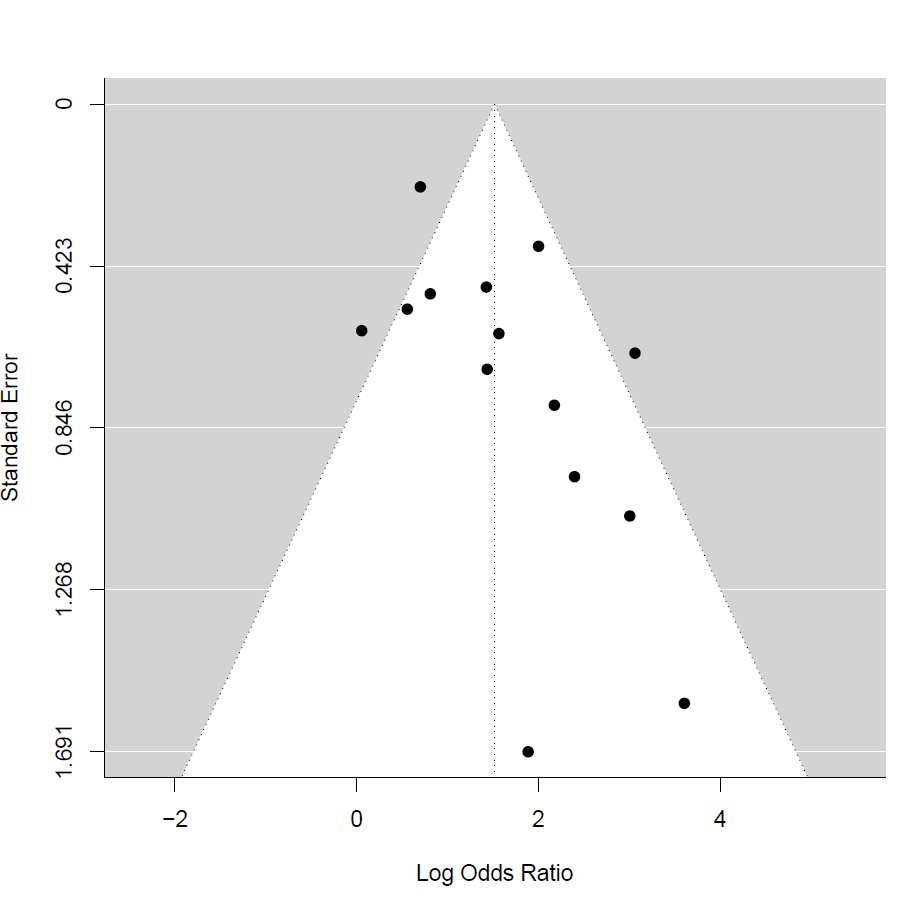


Figure S4. Diagnostic plot evaluating distribution of the 14 studies for association between hydrocephalus and functional outcomes (p-value for asymmetrical distribution, 0.034).


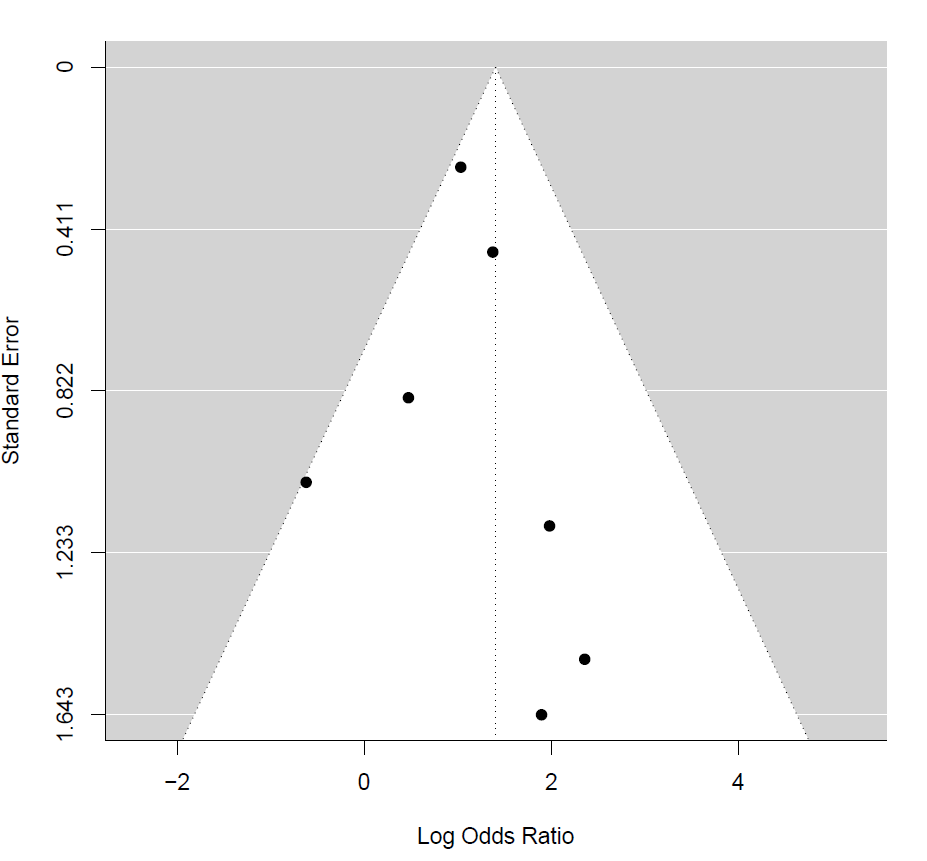


Figure S5. Diagnostic plot evaluating distribution of the 14 studies for association between abnormal brain MRI and functional outcomes (p-value for asymmetrical distribution, 0.842).


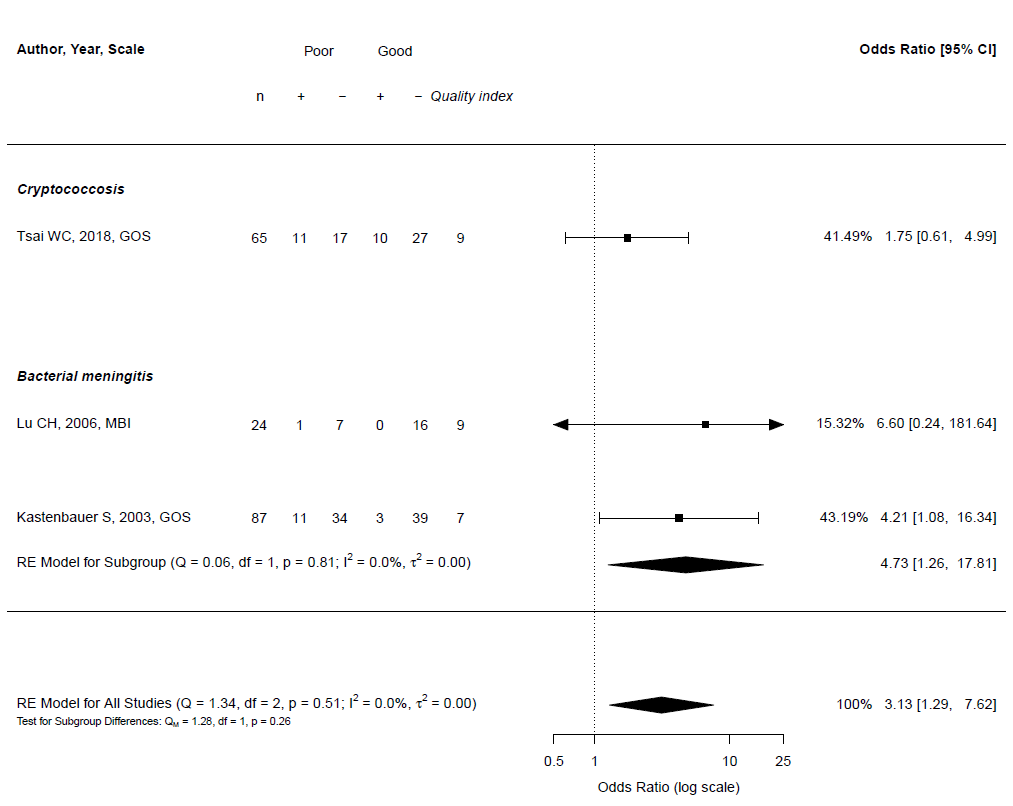
Figure S6. Forest plot illustrating the relationship between hydrocephalus on MRI in all infections except tuberculosis and unfavorable outcomes across included studies. “Quality index” refers to the quality scale adapted from NOS and RoBANS
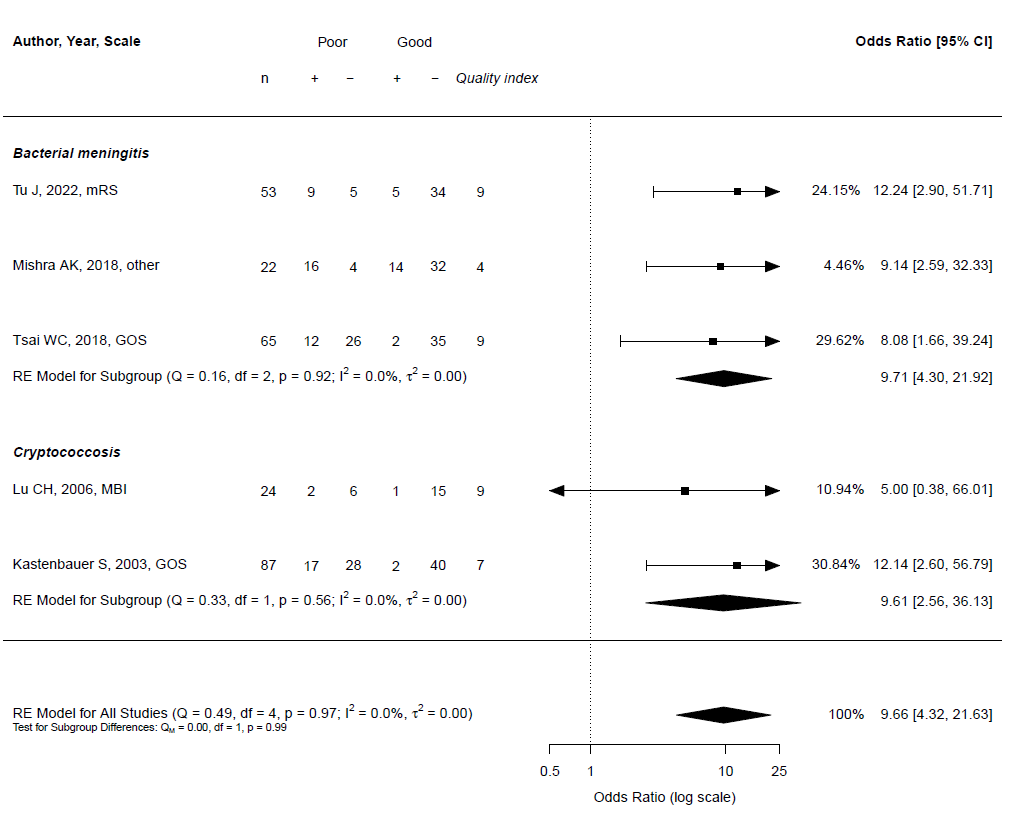


Figure S7. Forest plot illustrating the relationship between brain infarction on MRI in all infections except tuberculosis and unfavorable outcomes across included studies. “Quality index” refers to the quality scale adapted from NOS and RoBANS.


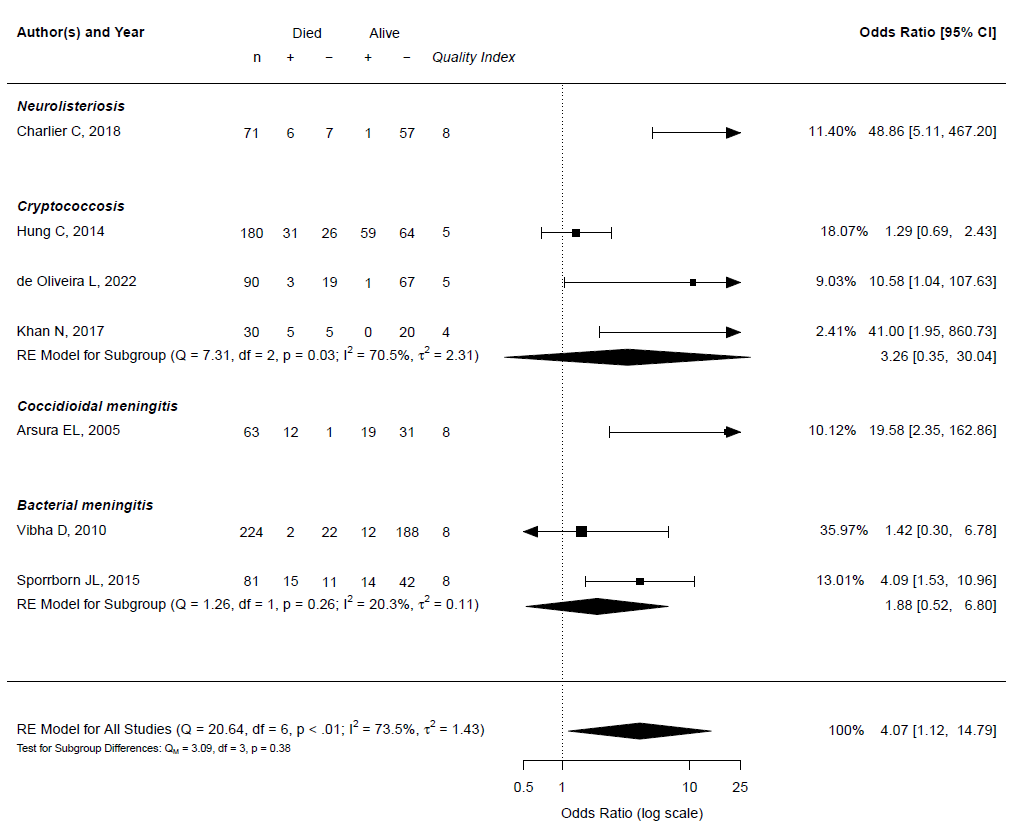


Figure S8. Forest plot illustrating the relationship between hydrocephalus on MRI in all infections except tuberculosis and mortality across included studies. “Quality index” refers to the quality scale adapted from NOS and RoBANS.


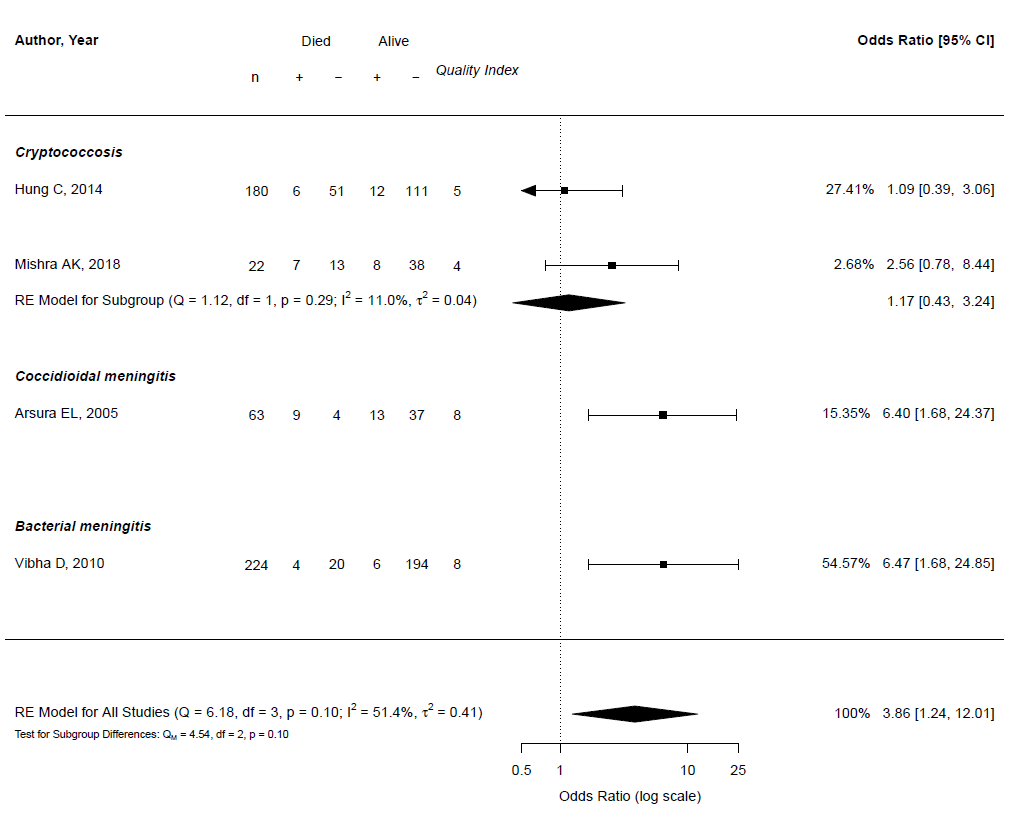


Figure S9 Forest plot illustrating the relationship between brain infarction on MRI in all infections except tuberculosis and mortality across included studies. “Quality index” refers to the quality scale adapted from NOS and RoBANS.


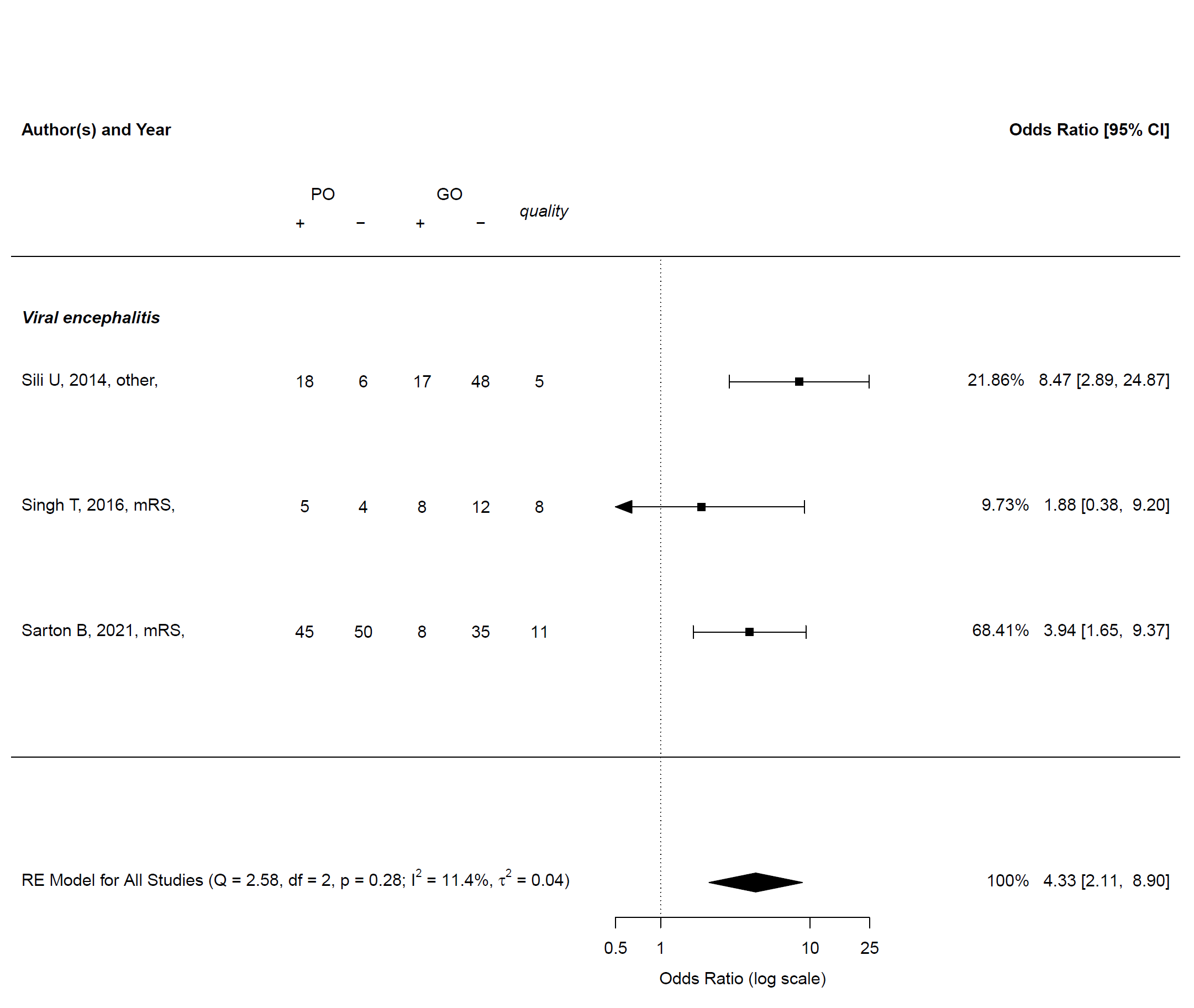


Figure S10 Forest plot illustrating the relationship between bilateral involvement on MRI in viral encephalitis and unfavorable outcomes across included studies. “Quality index” refers to the quality scale adapted from NOS and RoBANS.


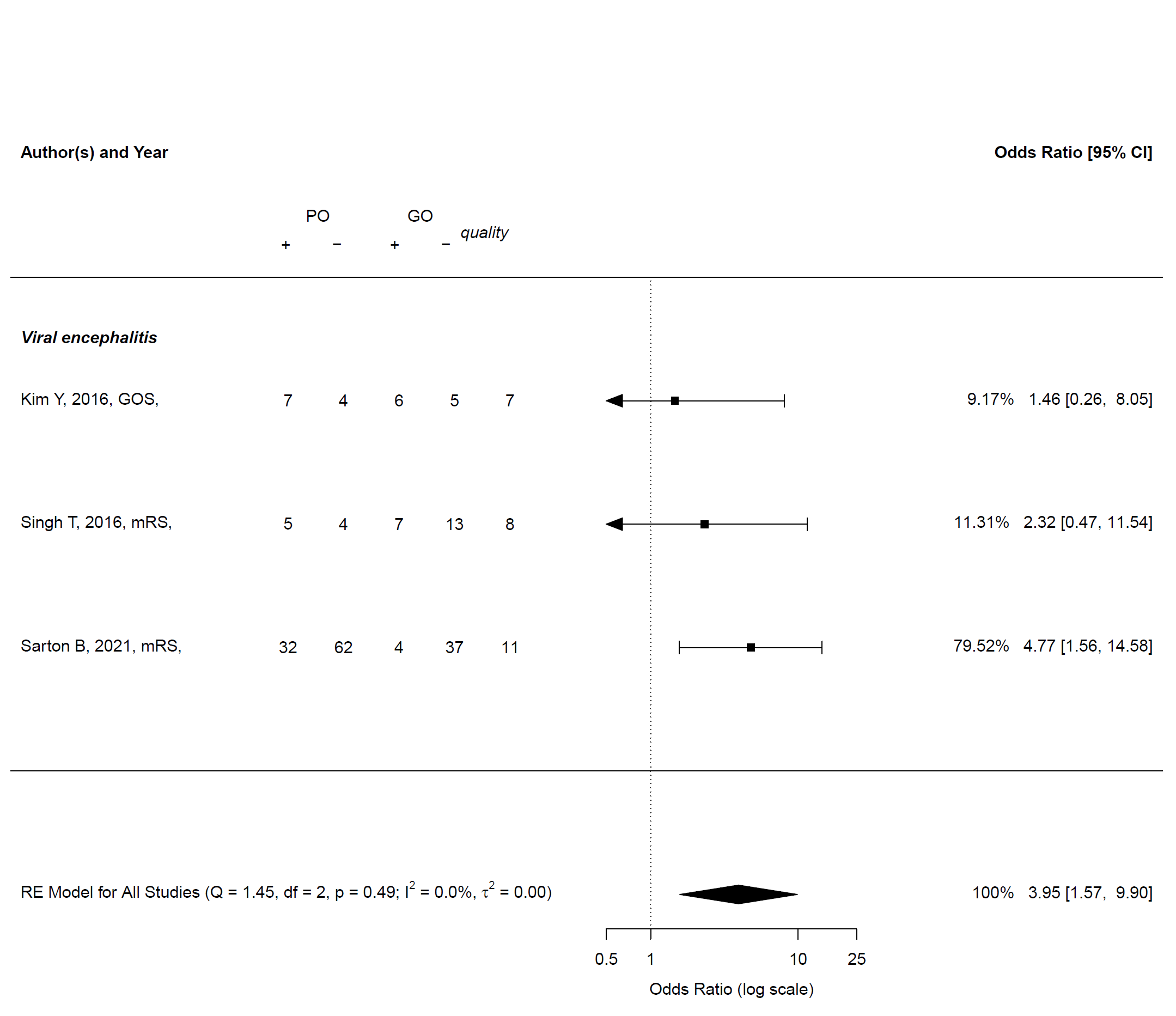


Figure S11 Forest plot illustrating the relationship between restricted diffusion on MRI in viral encephalitis and unfavorable outcomes across included studies. “Quality index” refers to the quality scale adapted from NOS and RoBANS.


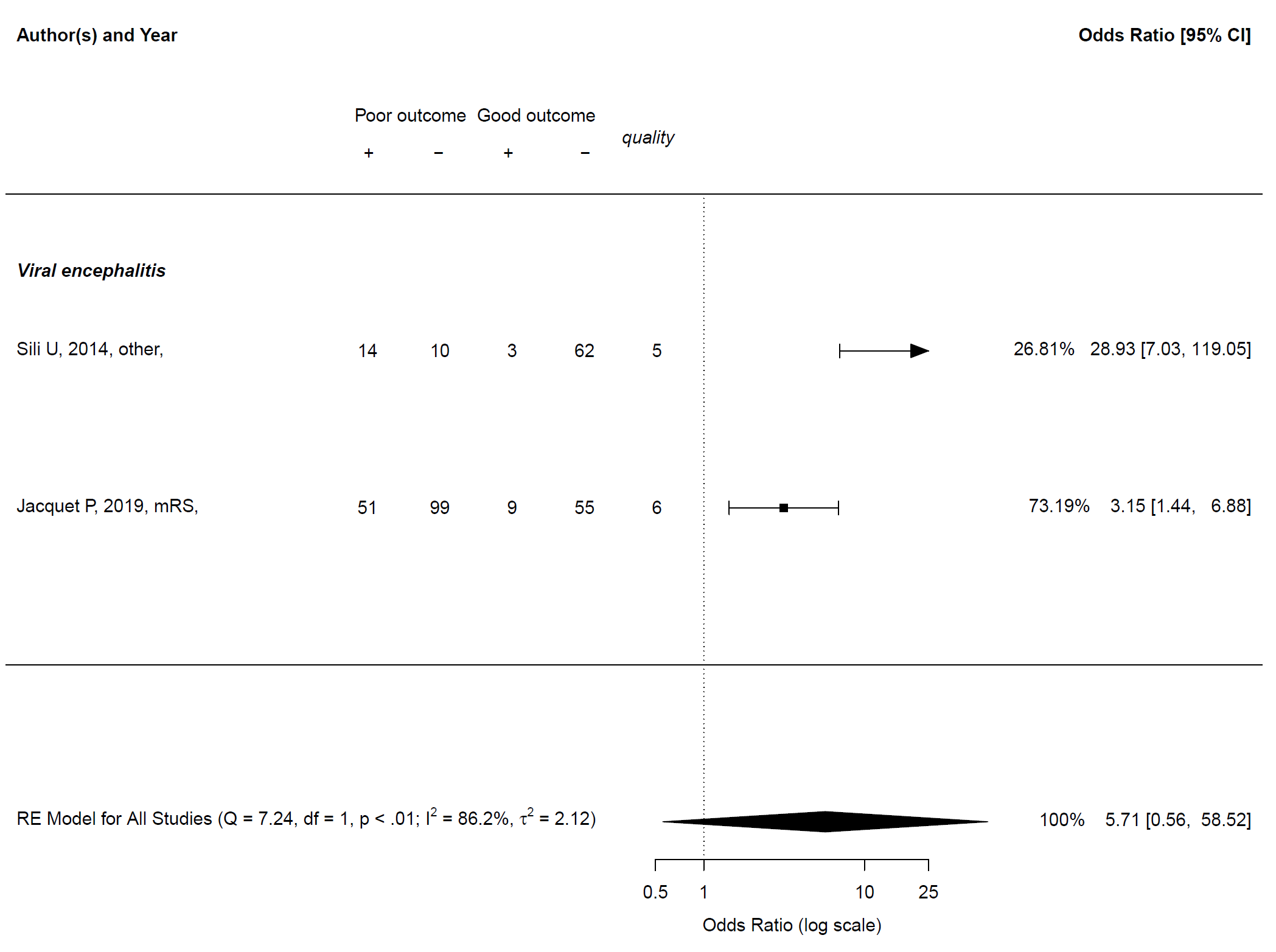
Figure S12 Forest plot illustrating the relationship between extensive involvement on MRI in viral encephalitis and unfavorable outcomes across included studies. “Quality index” refers to the quality scale adapted from NOS and RoBANS.


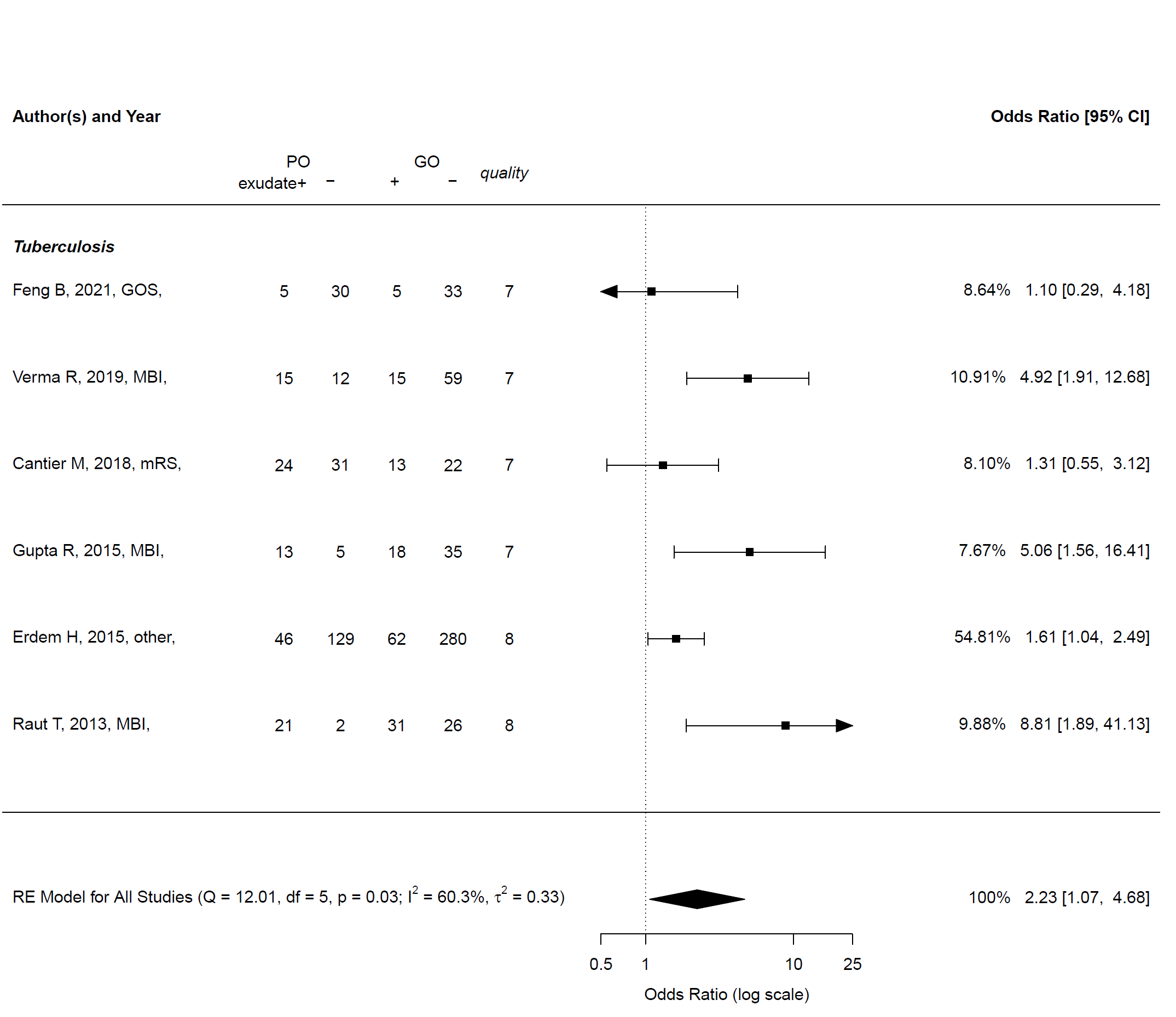


Figure S13 Forest plot illustrating the relationship between basal exudates on MRI in neuromeningeal tuberculosis and unfavorable outcomes across included studies. “Quality index” refers to the quality scale adapted from NOS and RoBANS.


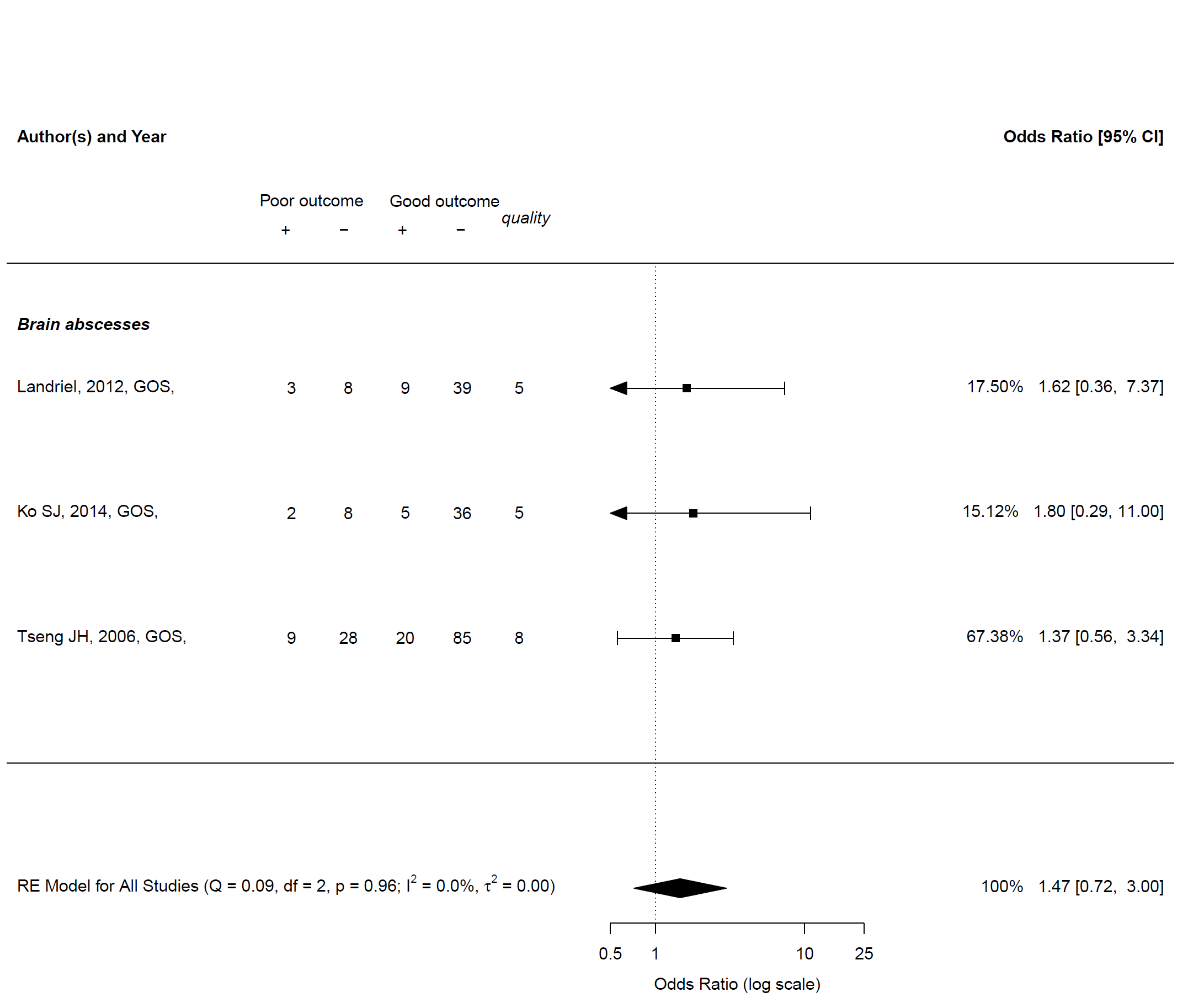


Figure S14 Forest plot illustrating the relationship between multiple location in brain abscesses and unfavorable outcomes across included studies. “Quality index” refers to the quality scale adapted from NOS and RoBANS.


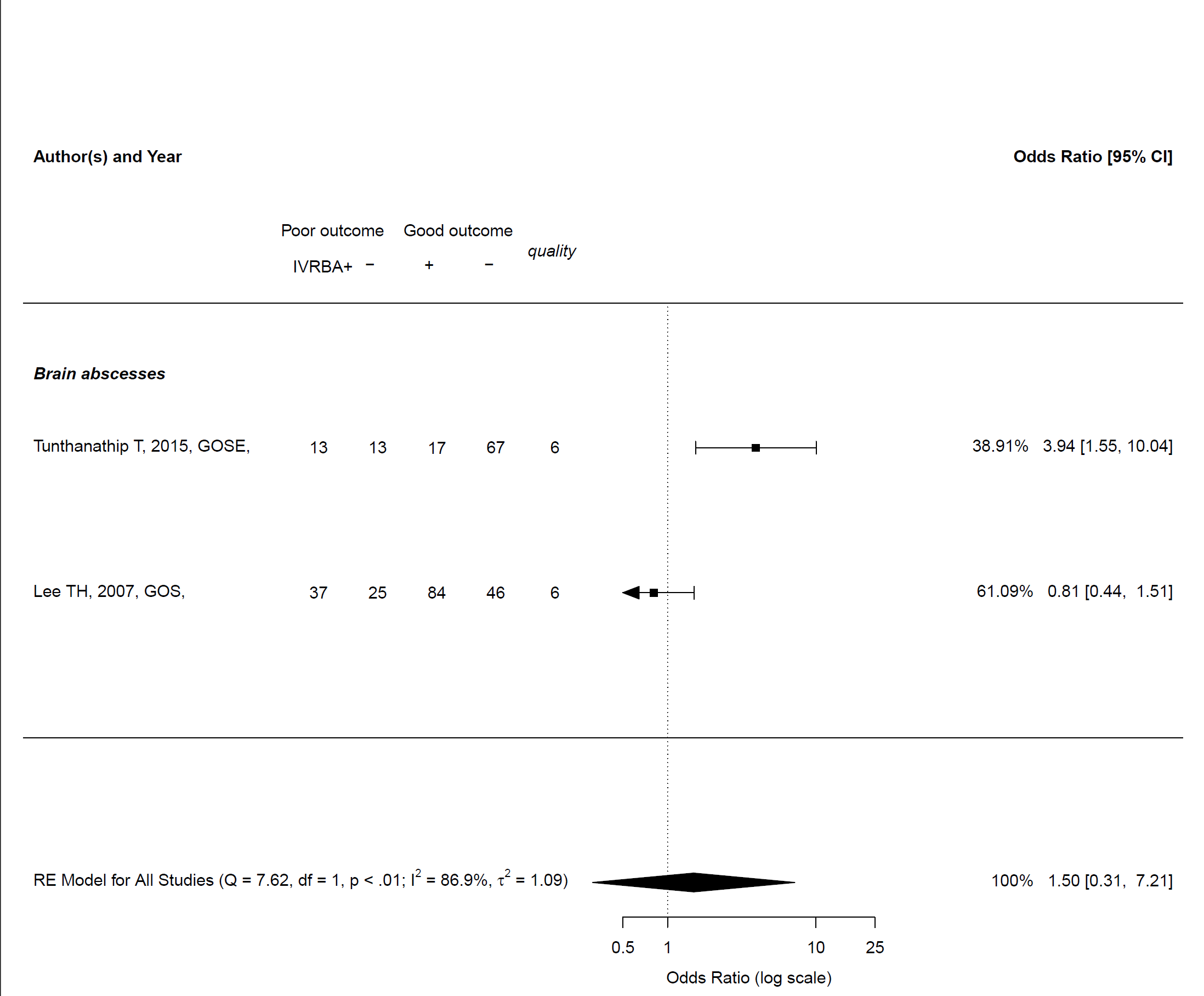


Figure S15 Forest plot illustrating the relationship between intraventricular rupture in brain abscesses (IVRBA) and unfavorable outcomes across included studies. “Quality index” refers to the quality scale adapted from NOS and RoBANS.


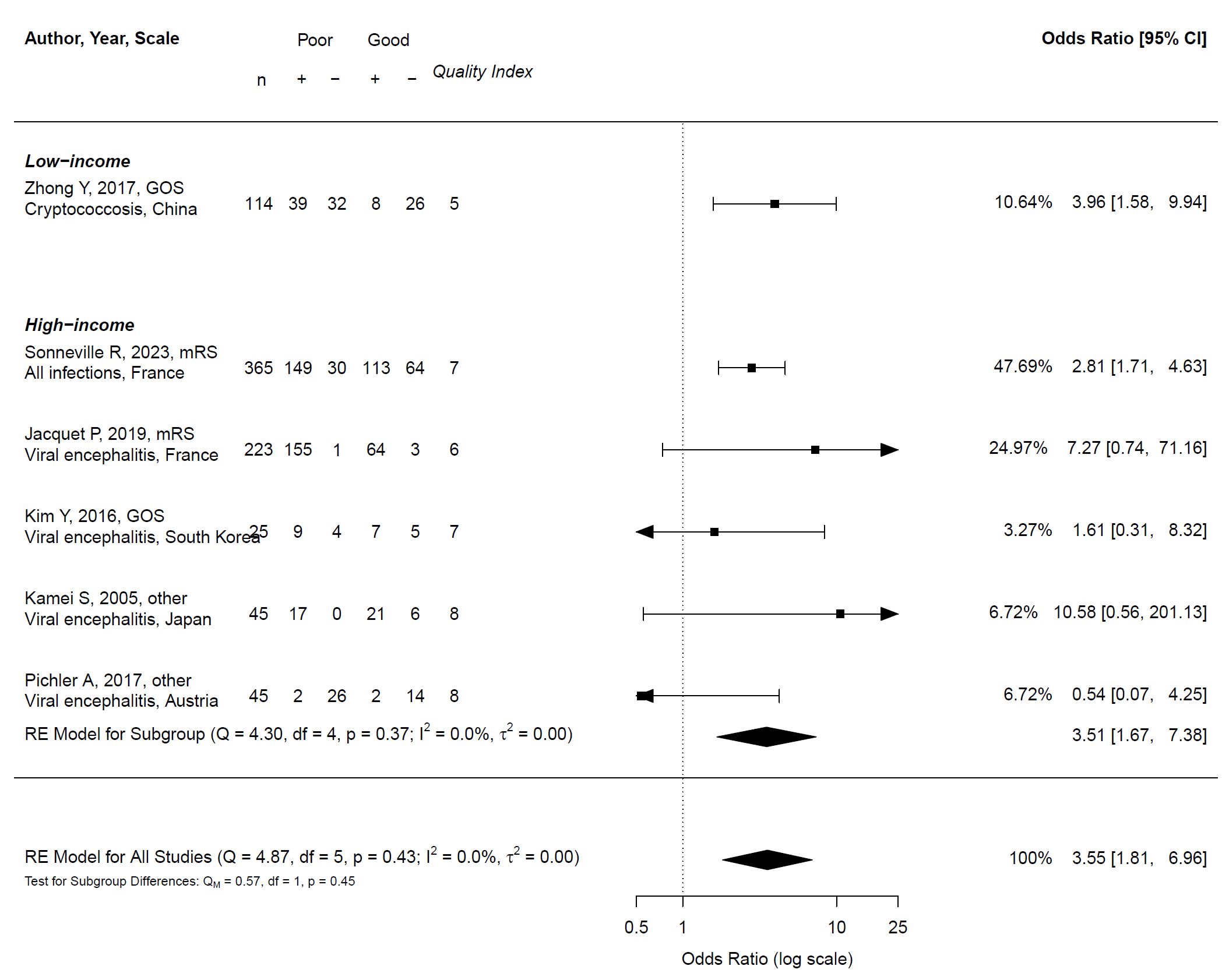


Figure S16. Forest plot illustrating the relationship between abnormal MRI and functional outcome across all included studies, with a subgroup analysis based on national income. "Quality index" refers to the quality scale adapted from NOS and RoBANS.


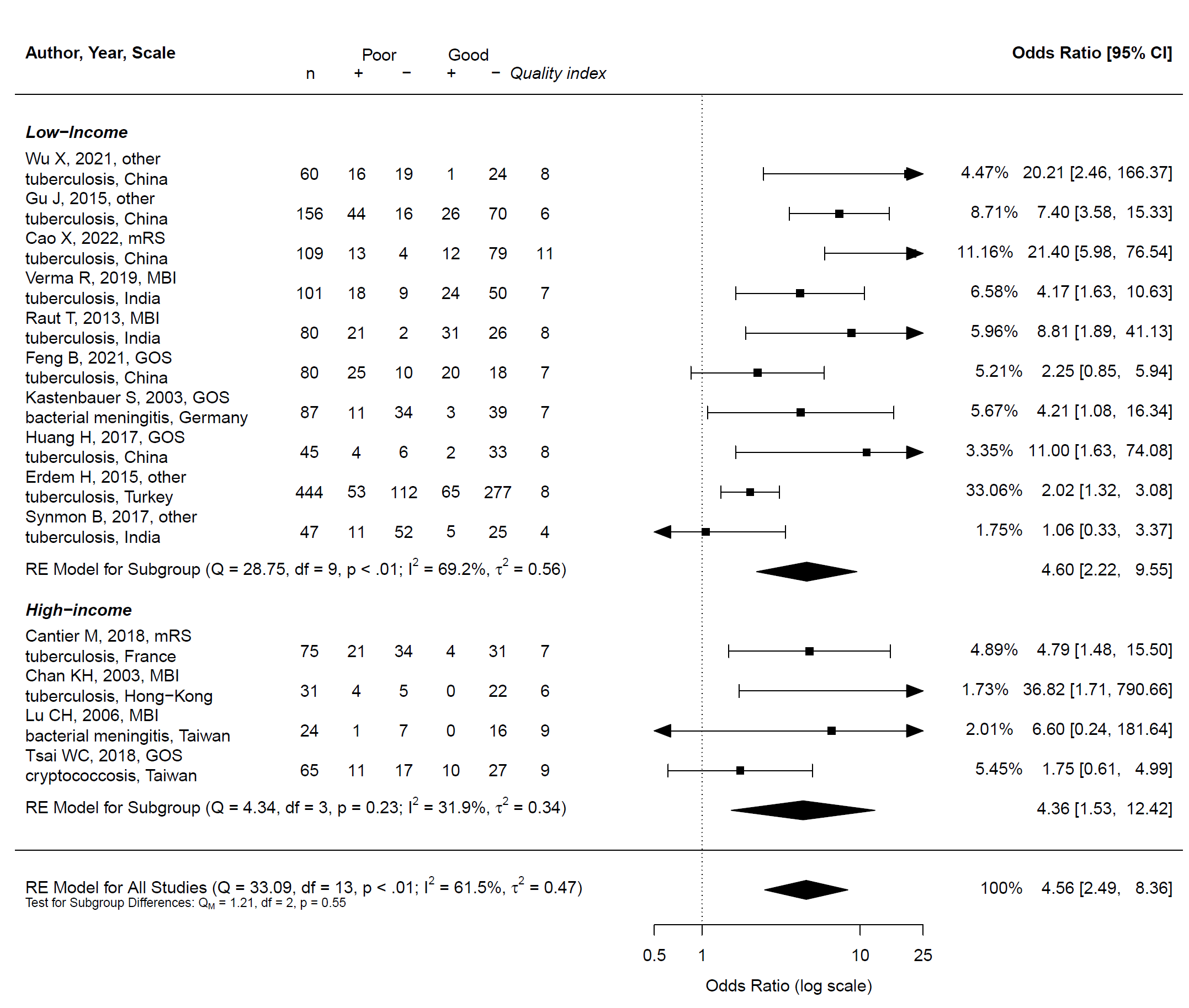
 Figure S17. Forest plot illustrating the relationship between hydrocephalus and functional outcome across all included studies, with a subgroup analysis based on national income. "Quality index" refers to the quality scale adapted from NOS and RoBANS.


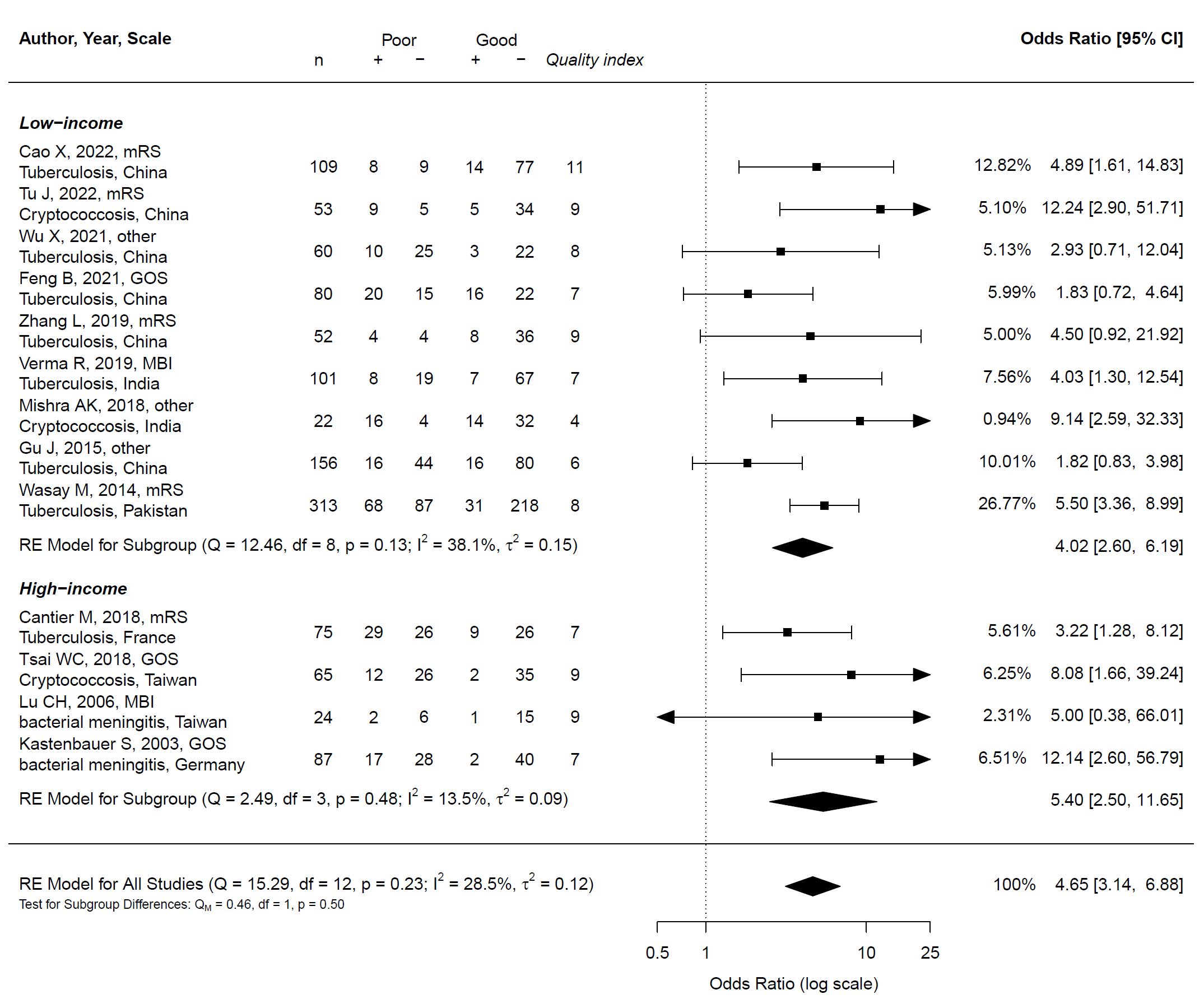


Figure S18. Forest plot illustrating the relationship between brain ischemia and functional outcome across all included studies, with a subgroup analysis based on national income. "Quality index" refers to the quality scale adapted from NOS and RoBANS.


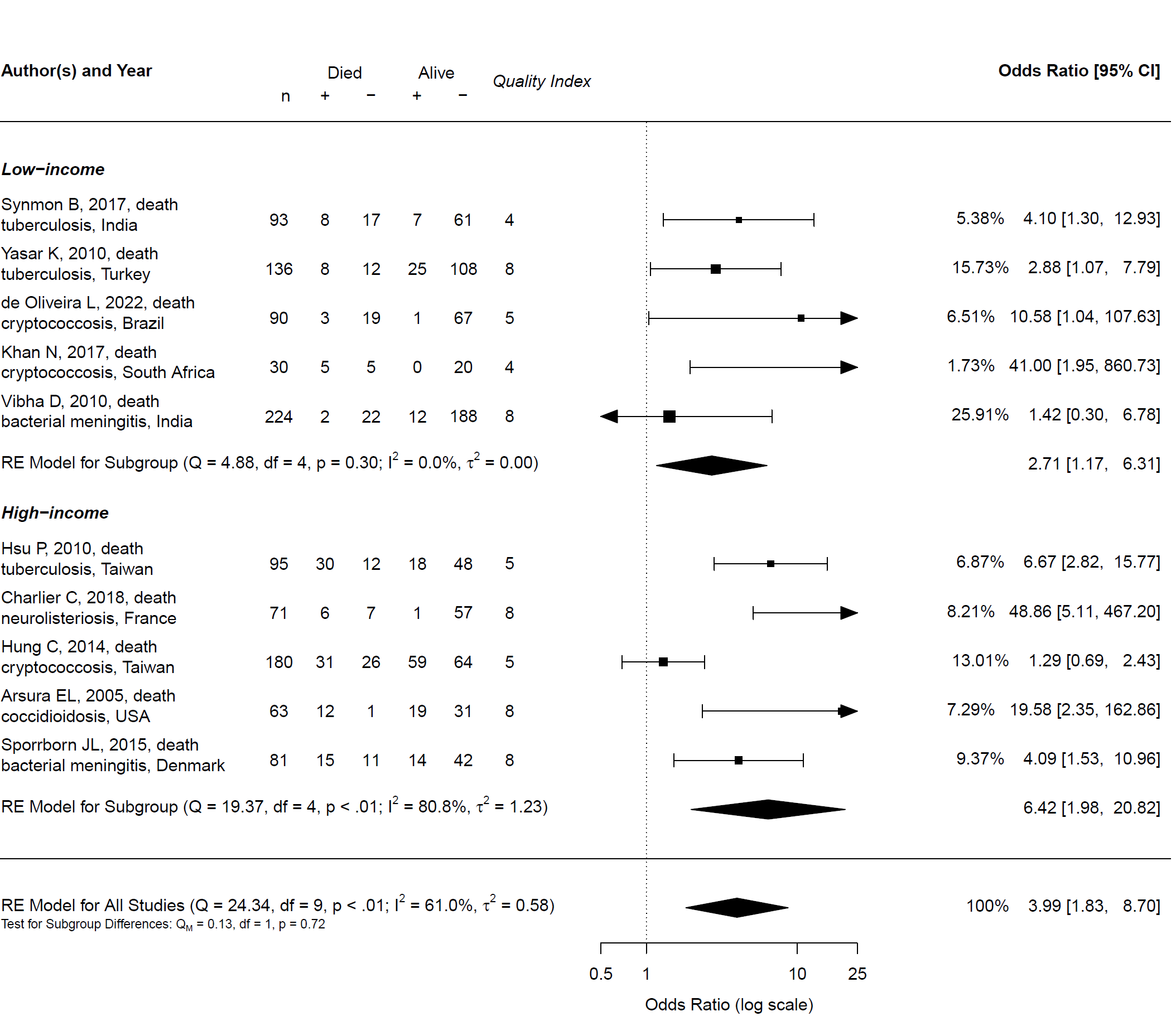


Figure S19. Forest plot illustrating the relationship between hydrocephalus and mortality across all included studies, with a subgroup analysis based on national income. "Quality index" refers to the quality scale adapted from NOS and RoBANS.


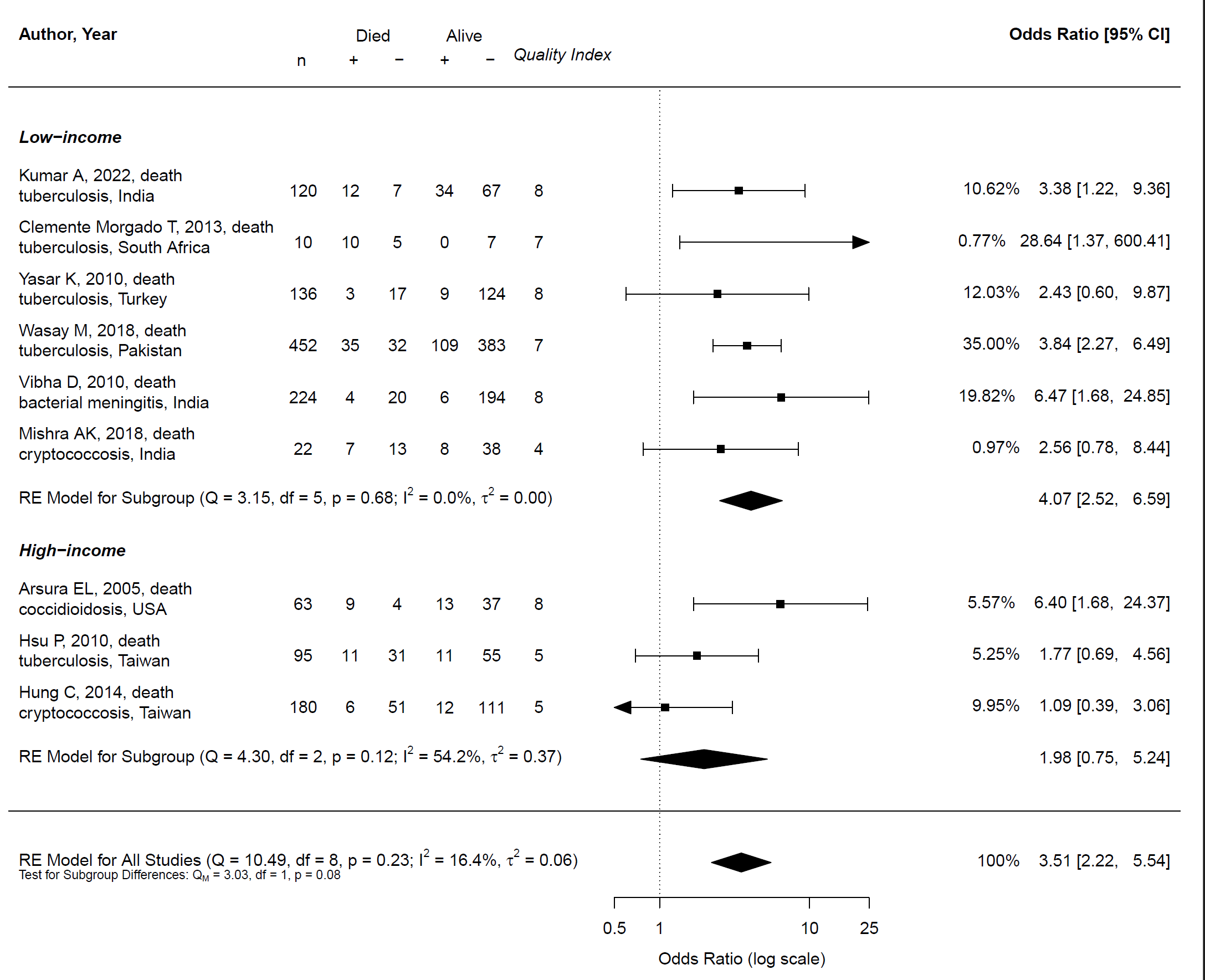


Figure S20. Forest plot illustrating the relationship between brain infarction and mortality across all included studies, with a subgroup analysis based on national income. "Quality index" refers to the quality scale adapted from NOS and RoBANS.


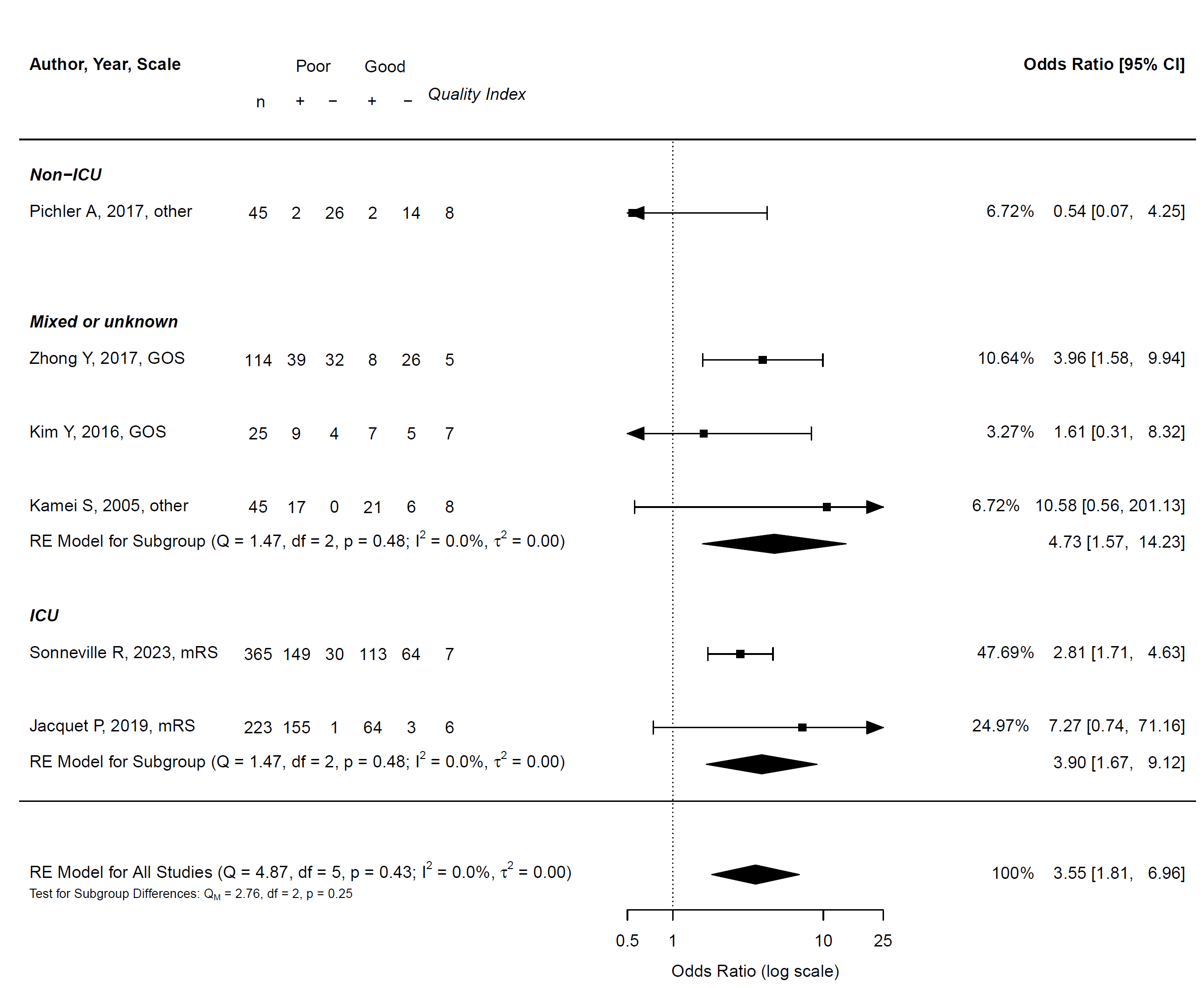
Figure S21. Forest plot illustrating the relationship between abnormal MRI and functional outcome across all included studies, with subgroup analysis based on the patient's hospitalization ward. "Quality index" refers to the quality scale adapted from NOS and RoBANS.


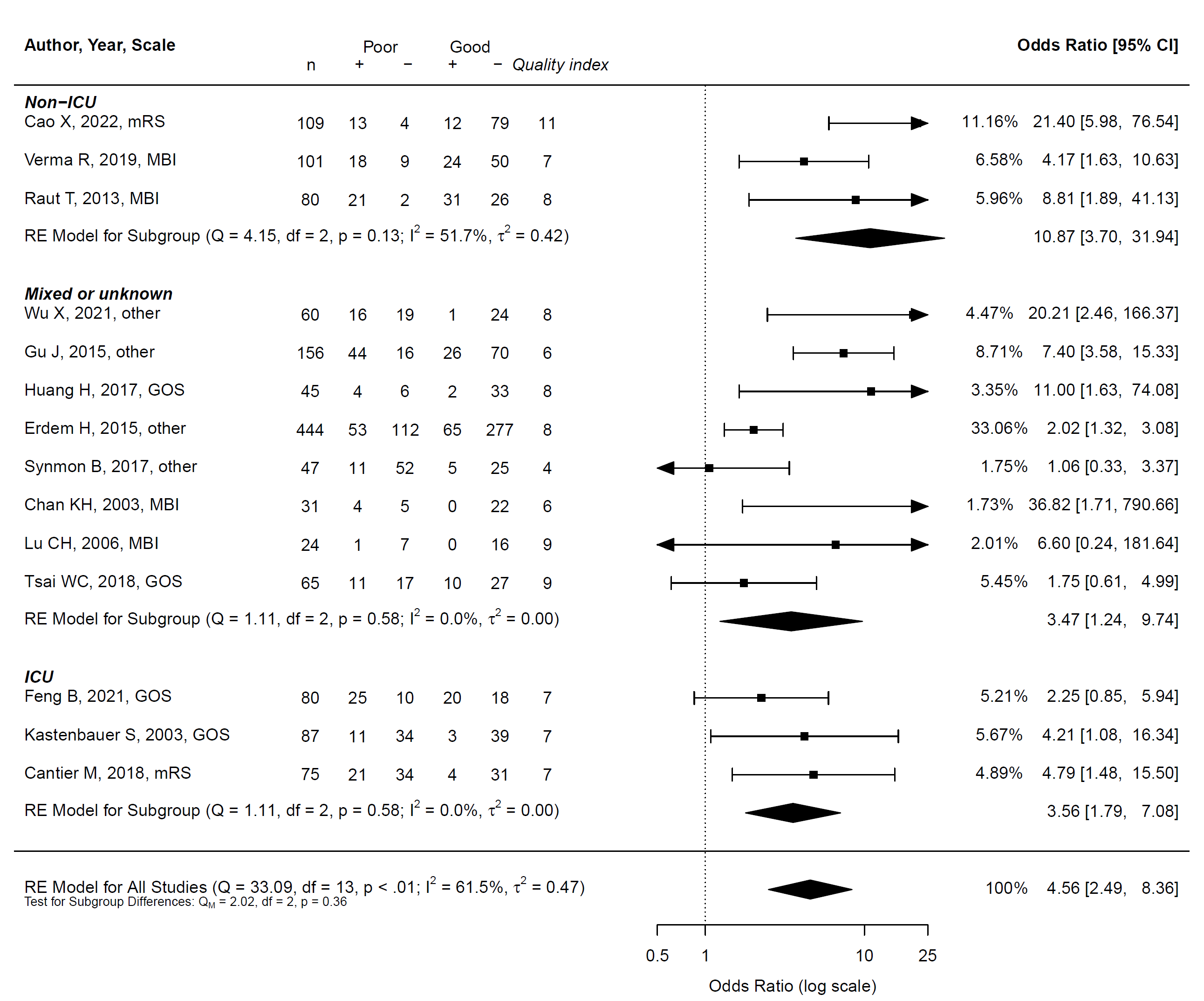


Figure S22. Forest plot illustrating the relationship between hydrocephalus and functional outcome across all included studies, with subgroup analysis based on the patient's hospitalization ward. "Quality index" refers to the quality scale adapted from NOS and RoBANS.


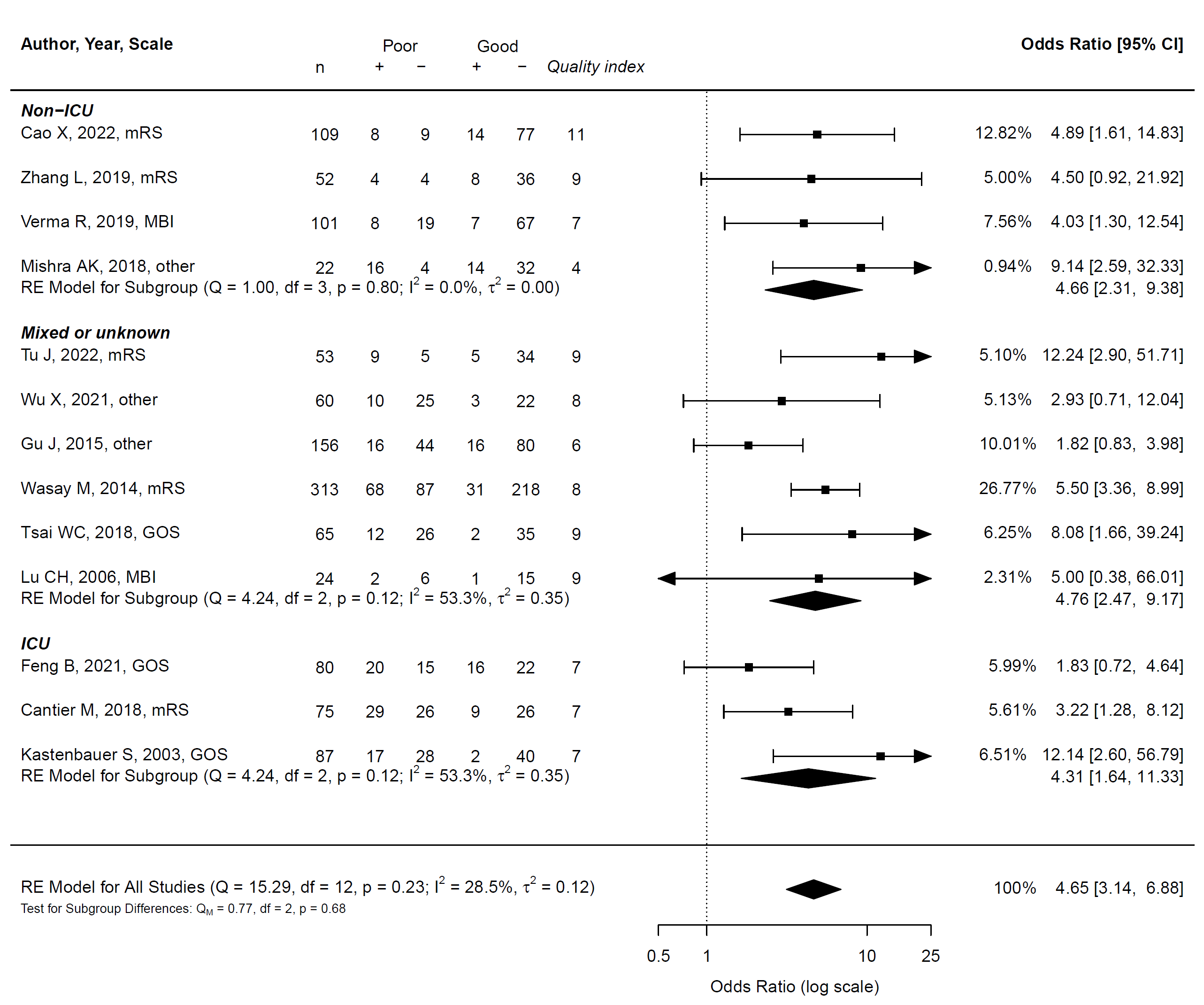


Figure S23. Forest plot illustrating the relationship between brain ischemia and functional outcome across all included studies, with subgroup analysis based on the patient's hospitalization ward. "Quality index" refers to the quality scale adapted from NOS and RoBANS.



Figure S24. Forest plot illustrating the relationship between hydrocephalus and functional outcome across all included studies, with subgroup analysis based on imaging modality. "Quality index" refers to the quality scale adapted from NOS and RoBANS. No significant difference was found between modalities.





Figure S25. Forest plot illustrating the relationship between brain ischemia and functional outcome across all included studies, with subgroup analysis based on imaging modality. "Quality index" refers to the quality scale adapted from NOS and RoBANS. No significant difference was found between modalities.
